# Synthesis, characterization, biological activities, and computational studies of pyrazolyl–thiazole derivatives of thiophene†

**DOI:** 10.1039/d4ra06228k

**Published:** 2024-12-10

**Authors:** Seema K. Bhagwat, Tushar Janardan Pawar, Sayali A. Kulkarni, Amar A. Patil, Rahul Ashokrao More, J. Oscar C. Jimenez-Halla, Juan Andres Alvarado-Salazar, Jose Luis Olivares-Romero, Ghazala Muteeb, Enrique Delgado-Alvarado, Sachin V. Patil

**Affiliations:** a Department of Chemistry, Research Centre HPT Arts and RYK Science College (Affiliated to S. P. Pune University) Nashik 422005 Maharashtra India; b Red de Estudios Moleculares Avanzados, Campus III, Instituto de Ecología, A. C. Carretera Antigua a Coatepec 351 91073 Xalapa Veracruz Mexico; c Department of Microbiology, Dayanand Science College Latur 413512 Maharashtra India; d Departamento de Química, División de Ciencias Naturales y Exactas, Universidad de Guanajuato Noria Alta S/N 36050 Guanajuato Mexico; e Carrera de Química-Farmacéutico-Biológica, Facultad de Estudios Superiores Zaragoza, UNAM 09230 Ciudad de México Mexico; f Department of Nursing, College of Applied Medical Sciences, King Faisal University Al-Ahsa Saudi Arabia; g Micro and Nanotechnology Research Center, Universidad Veracruzana Blvd. Av. Ruiz Cortines No. 455 Fracc. Costa Verde Boca del Río Veracruz 94294 Mexico

## Abstract

This study reports the synthesis, characterization, and biological evaluation of a series of pyrazolyl–thiazole derivatives of thiophene. Seven compounds were synthesized and characterized using NMR spectroscopy and mass spectrometry. The antimicrobial activities of these derivatives were evaluated against various bacterial (*Escherichia coli*, *Bacillus subtilis*, *Bacillus megaterium*, *Staphylococcus aureus*) and fungal strains (*Aspergillus niger*, *Aspergillus oryzae*, *Rhizopus*, *Candida albicans*), demonstrating significant inhibition zones and low minimum inhibitory concentrations (MIC). In addition, the compounds exhibited notable antioxidant activities in DPPH and hydroxyl radical scavenging assays. Computational studies, including density functional theory (DFT) calculations and molecular docking simulations, were performed to understand the electronic properties and binding interactions of the synthesized compounds with biological targets. The molecular docking results supported the experimental findings, highlighting the potential of these pyrazolyl–thiazole derivatives as multifunctional therapeutic agents with both antimicrobial and antioxidant properties.

## Abstract 2

This study reports the synthesis, characterization, computational studies and biological evaluation of a series of pyrazolyl–thiazole derivatives of thiophene.

## Graphical abstract

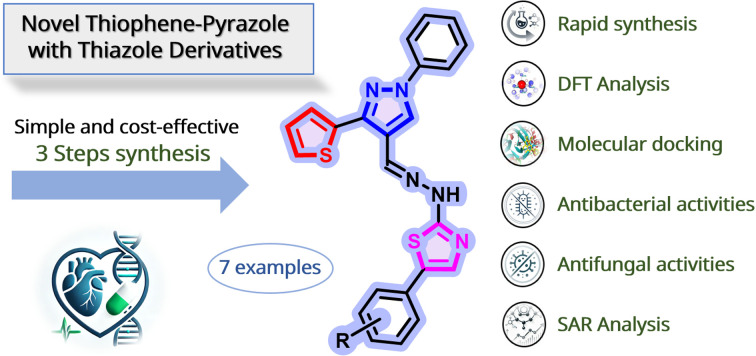

## Introduction

The emergence of drug-resistant pathogens and the increasing prevalence of oxidative stress-related diseases have underscored the urgent need for new and effective antimicrobial and antioxidant agents. Antimicrobial resistance poses a significant global health challenge, as many conventional antibiotics are becoming less effective against resistant strains.^1^ This crisis has spurred the exploration of novel chemical entities capable of combating these resilient pathogens. Heterocyclic compounds have been recognized for their potential in medicinal chemistry due to their versatile chemical structures and diverse biological activities.^2^ Among these, thiazole, pyrazole and thiophene derivatives have garnered significant interest for their antimicrobial and antioxidant properties. Additionally, the combination of peptides with these heterocyclic compounds is an emerging area of research, offering new opportunities to develop hybrid molecules with enhanced biological activities and reduced toxicity.^3^

Thiazole, pyrazole, and thiophene are three important five-membered heterocyclic rings widely recognized for their significant roles in medicinal chemistry. Thiazole, containing both sulfur and nitrogen atoms, is found in many bioactive molecules with antimicrobial, anti-inflammatory, and anticancer properties.^4^ It serves as a core structure in compounds like vitamin B1 (thiamine) and the antibiotic bacitracin, highlighting its importance in biological systems and drug design.^5^ Thiazole derivatives have demonstrated substantial antimicrobial activity against a wide range of pathogens, making them promising scaffolds for developing new antibiotics. Pyrazole, another five-membered ring with two adjacent nitrogen atoms, also exhibits diverse pharmacological activities, including anti-inflammatory, analgesic, antipyretic, and antitumor effects.^6^ The therapeutic potential of pyrazoles is underscored by their presence in well-known drugs such as celecoxib, an anti-inflammatory agent, and sildenafil, used to treat erectile dysfunction. Pyrazole rings are particularly valuable in drug design due to their ability to modulate enzyme activity, providing a basis for developing enzyme inhibitors for various diseases.^7^

Thiophene, a five-membered ring containing sulfur, is another versatile heterocyclic compound that has garnered attention for its biological activities. Thiophene derivatives are known for their antimicrobial, anti-inflammatory, and antitumor properties.^8^ They are often incorporated into drugs and biologically active molecules due to their ability to enhance the pharmacokinetic and pharmacodynamic profiles of these compounds. The presence of sulfur in thiophene contributes to its unique electronic properties, which can improve binding affinity and specificity towards biological targets. The combination of thiophene with other heterocyclic rings, such as thiazole and pyrazole, can lead to hybrid molecules with enhanced biological activities and therapeutic potential.

Together, thiazole, pyrazole, and thiophene represent a diverse group of heterocyclic compounds with broad-spectrum biological activities. Their ability to interact with various biological targets, combined with their versatile chemical structures, makes them invaluable in the development of new drugs. By exploring the potential of these rings, researchers can design hybrid molecules that leverage the unique properties of each to create more effective and targeted therapeutic agents.^9^

While thiophenyl–pyrazolyl–thiazole hybrids have been explored for their antimicrobial and DHFR inhibitory activities, particularly against *Mycobacterium tuberculosis*, our study presents a distinct approach by synthesizing a new series of these derivatives with unique structural modifications. Unlike previous studies, including the recent work by Dawood *et al.* (2023),^10^ which focused on DHFR inhibition and antimicrobial activity, our research broadens the biological scope to include antioxidant activities and explores different biological targets. Additionally, we employed advanced computational techniques to predict electronic properties and stability, providing new insights into the structure–activity relationship. These differences in chemical structure, biological evaluation, and computational analysis highlight the novelty of our work and its potential contribution to developing multifunctional agents with diverse therapeutic applications.

In this study, we report the synthesis, characterization, and biological evaluation of a novel series of pyrazolyl–thiazole derivatives of thiophene, which have not been previously explored in the literature. Unlike existing studies that focus primarily on individual heterocyclic rings, our work investigates hybrid molecules combining thiazole, pyrazole, and thiophene scaffolds. This unique combination is designed to exploit the complementary pharmacological properties of these rings, leading to enhanced antimicrobial and antioxidant activities. Additionally, our work incorporates comprehensive computational studies, including DFT calculations and molecular docking, to provide a deeper understanding of the electronic properties and binding interactions of these novel compounds. The insights gained from these computational studies offer valuable guidance for the rational design of more potent derivatives.

## Results and discussion

### Synthesis and characterization of pyrazolyl–thiazole derivatives

**Scheme 1:**
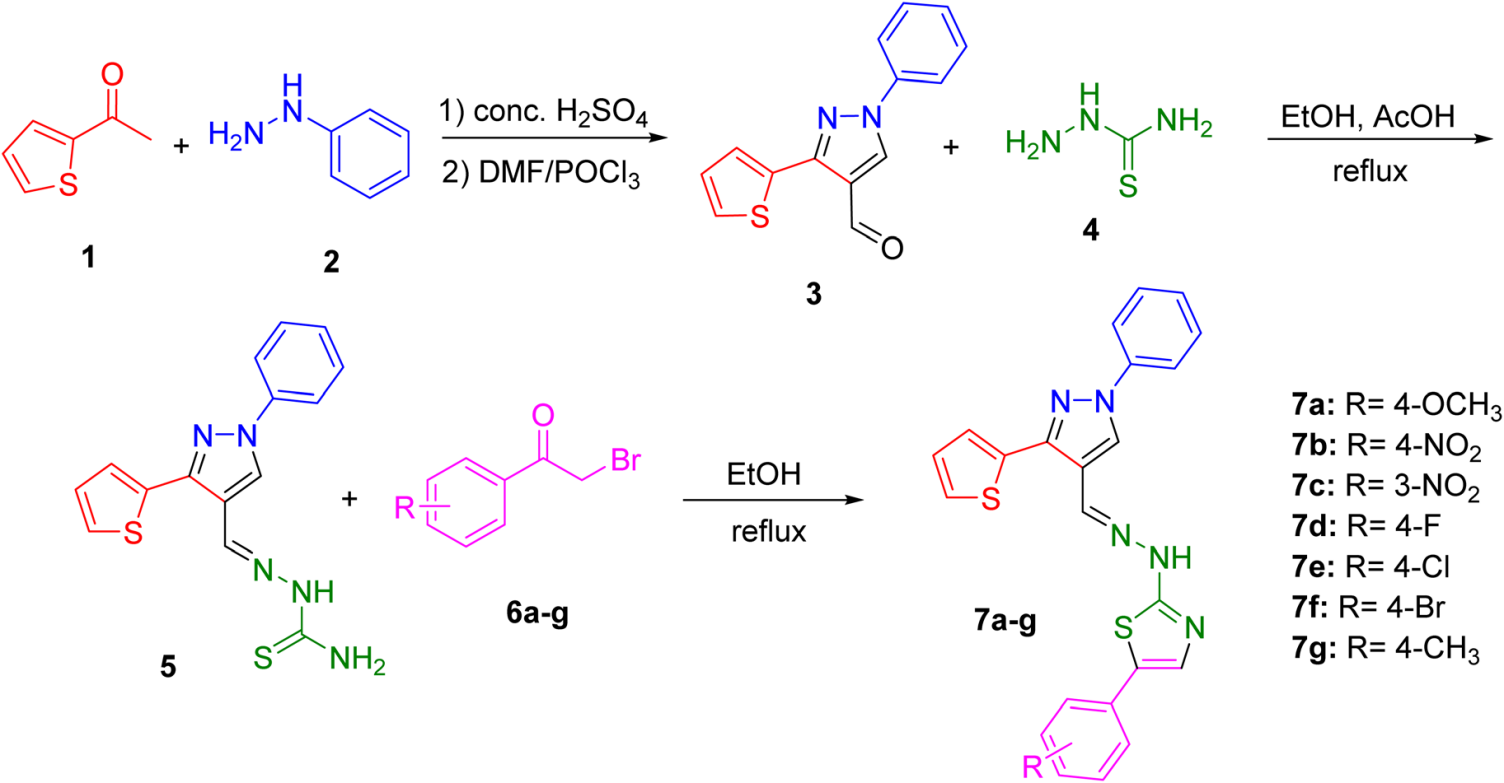
Synthetic route for the pyrazolyl–thiazole derivatives.

The synthesis of the pyrazolyl–thiazole derivatives of thiophene was carried out through a multi-step synthetic route, as outlined in Scheme 1. Using previously reported procedure,^11^ the process began with the condensation of acetyl thiophene 1 with phenyl hydrazine 2 in the presence of concentrated H_2_SO_4_. This reaction yielded the hydrazone intermediate, which was then cyclized using phosphoryl chloride (POCl_3_) in dimethylformamide (DMF) to form the desired pyrazole-4-carbaldehyde 3. The product was purified by recrystallization from ethanol. Next, the pyrazole-4-carbaldehyde 3 was reacted with thiosemicarbazide 4 in ethanol with acetic acid as a catalyst to form the thiosemicarbazone derivative 5. The reaction mixture was refluxed for one hour. Upon completion, the mixture was cooled to room temperature, and the solid product was filtered, washed with ethanol, and recrystallized. The final step involved the reaction of the thiosemicarbazone intermediate 5 with various substituted phenacyl bromides 6a–g in ethanol under reflux conditions. This reaction produced the final pyrazolyl–thiazole derivatives 7a–g, which were filtered, washed with ethanol, and recrystallized to obtain pure products. The synthesized derivatives included compounds with different substituents on the phenyl ring: 7a (R = 4-OCH_3_), 7b (R = 4-NO_2_), 7c (R = 3-NO_2_), 7d (R = 4-F), 7e (R = 4-Cl), 7f (R = 4-Br), and 7g (R = 4-CH_3_).

The structures of the compounds 7a–g were confirmed by NMR spectroscopy and mass spectrometry. The ^1^H and ^13^C NMR spectra displayed characteristic signals corresponding to the pyrazole, thiazole, and thiophene moieties, as well as the substituents on the phenyl ring. Mass spectrometry provided molecular ion peaks that matched the expected molecular weights of the compounds 7a–g, confirming their molecular formulas.

### Antimicrobial activity

The antimicrobial activities of the synthesized pyrazolyl–thiazole derivatives 7b–g were evaluated against a panel of bacterial and fungal strains. The bacterial strains tested included *Escherichia coli*, *Bacillus subtilis*, *Bacillus megaterium*, and *Staphylococcus aureus*. The fungal strains tested included *Aspergillus niger*, *Aspergillus oryzae*, *Rhizopus*, and *Candida albicans*.

**Fig. 1:**
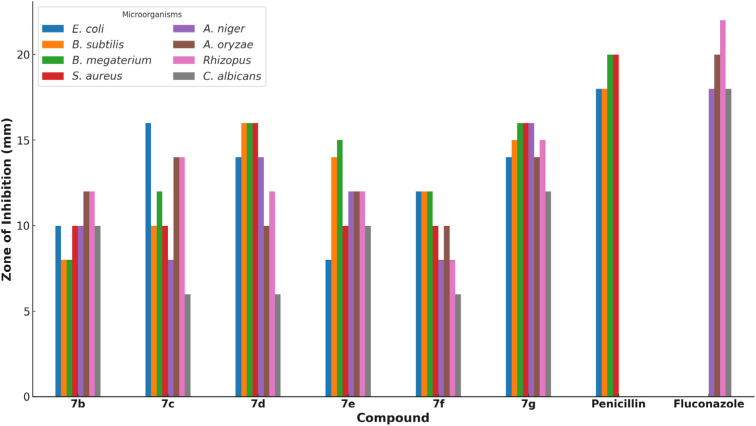
Zone of inhibition (mm) of compounds 7b–g and standard antibiotics (penicillin, fluconazole) against various bacterial (*Escherichia coli*, *Bacillus subtilis*, *Bacillus megaterium*, *Staphylococcus aureus*) and fungal strains (*Aspergillus niger*, *Aspergillus oryzae*, *Rhizopus*, *Candida albicans*). The standard antibiotics penicillin and fluconazole are included as controls for comparison. Higher values indicate greater inhibition of microbial growth, reflecting the antimicrobial potency of each compound.

The antimicrobial activity of each compound was assessed using the Kirby–Bauer disk diffusion method. In this method, disks impregnated with the synthesized compounds were placed on agar plates inoculated with the respective microbial strains. The plates were then incubated, and the zones of inhibition around each disk were measured to determine the antimicrobial effectiveness of the compounds. The results are summarized in Table 1. Compounds 7c and 7d exhibited the highest antibacterial activity, particularly against *Bacillus subtilis* and *Bacillus megaterium*, with inhibition zones of 16 mm (Fig. 1). Compound 7g also showed significant activity with inhibition zones of 15–16 mm against multiple strains.

**Table 1:** Zone of inhibition of compounds 7b–g against bacterial and fungal strains. The zones were measured in millimeters using the Kirby–Bauer disk diffusion method. Larger inhibition zones indicate higher antimicrobial activity

Compound	*E. coli*	*B. subtilis*	*B. megaterium*	*S. aureus*	*A. niger*	*A. oryzae*	*Rhizopus*	*C. albicans*
7b	10	8	8	10	10	12	12	10
7c	16	10	12	10	8	14	14	6
7d	14	16	16	16	14	10	12	6
7e	8	14	15	10	12	12	12	10
7f	12	12	12	10	8	10	8	6
7g	14	15	16	16	16	14	15	12
Penicillin	18	18	20	20	—	—	—	—
Fluconazole	—	—	—	—	18	20	22	18

**Fig. 2:**
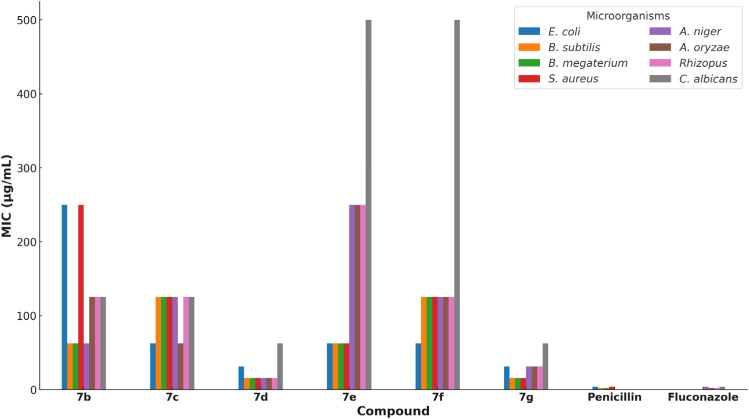
Minimum inhibitory concentration (MIC) values (μg mL^−1^) of compounds 7b–g and standard antibiotics (penicillin, fluconazole) against various bacterial and fungal strains. Lower MIC values indicate greater antimicrobial potency. Penicillin and fluconazole are included as standard controls.

In addition to the disk diffusion assay, the minimum inhibitory concentrations (MICs) of the synthesized compounds were determined using the resazurin microtiter assay (REMA). This assay provides quantitative data on the antimicrobial potency of the compounds by measuring the lowest concentration at which visible microbial growth is inhibited. The MIC values are presented in Table 2. Compounds 7d and 7g showed the lowest MIC values, indicating their potent antimicrobial activity (Fig. 2). Notably, compound 7d exhibited MIC values of 15.63 μg mL^−1^ against *Bacillus subtilis*, *Bacillus megaterium*, and various fungal strains, suggesting broad-spectrum antimicrobial efficacy.

**Table 2:** Minimum inhibitory concentration (MIC) of compounds 7b–g against bacterial and fungal strains (μg mL^−1^). MIC is defined as the lowest concentration of the compound required to inhibit visible microbial growth

Compound	*E. coli*	*B. subtilis*	*B. megaterium*	*S. aureus*	*A. niger*	*A. oryzae*	*Rhizopus*	*C. albicans*
7b	250	62.5	62.5	250	62.5	125	125	125
7c	62.5	125	125	125	125	62.5	125	125
7d	31.25	15.63	15.63	15.63	15.63	15.63	15.63	62.5
7e	62.5	62.5	62.5	62.5	250	250	250	500
7f	62.5	125	125	125	125	125	125	500
7g	31.25	15.63	15.63	15.63	31.25	31.25	31.25	62.5
Penicillin	3.9	1.95	1.95	3.9	—	—	—	—
Fluconazole	—	—	—	—	3.9	1.95	1.95	3.9

### Antioxidant activity

The antioxidant activities of the synthesized pyrazolyl–thiazole derivatives 7b–g were evaluated using two different assays: the DPPH (2,2-diphenyl-1-picrylhydrazyl) radical scavenging assay and the hydroxyl radical scavenging assay. These assays measure the ability of the compounds to neutralize free radicals, which are implicated in various diseases due to their role in oxidative stress.

#### DPPH radical scavenging activity

The DPPH radical scavenging assay is a widely used method to evaluate the free radical scavenging ability of compounds. In this assay, the ability of the synthesized compounds to donate hydrogen atoms or electrons to DPPH radicals was measured, resulting in a decrease in absorbance at 517 nm. The percent radical scavenging activity was calculated, and the results are summarized in Table 3. Compounds 7d and 7e exhibited the highest DPPH radical scavenging activity, with values of 69.4% and 72.45%, respectively. These activities are comparable to the standard antioxidant ascorbic acid.

**Table 3:** Free radical scavenging activity of compounds 7b–g expressed as the percentage inhibition of DPPH and hydroxyl radicals. Higher percentages indicate stronger antioxidant activity

Compound	DPPH (%)	Hydroxyl (%)
7b	58.45	65.45
7c	62.54	58.08
7d	69.40	64.00
7e	72.45	69.45
7f	62.57	58.41
7g	66.84	64.65
Ascorbic acid	81.64	—
α-Tocopherol	—	82.64

#### Hydroxyl radical scavenging activity

The hydroxyl radical scavenging assay assesses the ability of the compounds to scavenge hydroxyl radicals, which are highly reactive species that can cause severe damage to biomolecules. The percent hydroxyl radical scavenging activity was measured, and the results are presented in Table 3. Similar to the DPPH assay, compounds 7b, 7e, and 7g demonstrated the highest scavenging activities, indicating their potential as effective antioxidants.

### Computational studies

To further understand these observed biological activities, we conducted detailed computational studies. Our DFT calculations revealed variations in the HOMO–LUMO gaps among the compounds, with 7d and 7g exhibiting smaller gaps indicative of higher electronic stability and reactivity, which correlates with their enhanced antioxidant activity. Molecular docking studies identified key binding interactions between these compounds and biological targets, such as hydrogen bonding and π–π stacking, which align with the experimental antimicrobial data. Compounds 7d and 7g, which showed the most favorable binding affinities in docking studies, also demonstrated the highest antimicrobial activities *in vitro*. These findings underscore the utility of computational methods in predicting biological activity and guiding the design of more effective therapeutic agents.

#### Geometry optimization and electronic properties

To gain deeper insights into the electronic properties of the compounds, geometry optimizations were performed using density functional theory (DFT) at the B3LYP/6-31G(d) level of theory. Single-point energy calculations were conducted using ωB97X-D/def2-TZVPP to improve the accuracy of the electronic energy values. The calculations were performed using Gaussian 16 software. It should be noted that geometry optimizations were conducted in the gas phase, which may not fully account for solvent effects. However, given the molecular properties of the compounds, the gas-phase results are expected to provide a reasonable approximation. The optimized geometries provided detailed insights into the molecular conformations and bond lengths of the synthesized compounds. Notably, the pyrazolyl and thiazole rings were found to be nearly coplanar, which could enhance π–π interactions with biological targets. This coplanarity is expected to facilitate effective interactions with target proteins or enzymes. Key bond lengths and angles for each compound are provided in the ESI (pages S4–S13†), confirming that the obtained geometries are stable and correspond to energy minima.

#### HOMO–LUMO analysis

The energy gap between the Highest Occupied Molecular Orbital (HOMO) and the Lowest Unoccupied Molecular Orbital (LUMO), denoted as Δ*E*, was analysed to understand the chemical stability and reactivity of the compounds. The HOMO–LUMO energy values and gaps are summarized in Table 4. The Δ*E* values indicate that compounds with weak electron-withdrawing groups (7d–g) exhibit larger HOMO–LUMO gaps, suggesting greater chemical stability. In contrast, compounds with strong electron-withdrawing groups (7b–c) display smaller energy gaps, which correlate with enhanced reactivity and potential biological activity.

**Table 4:** Calculated frontier molecular orbitals and energy gap (Δ*E*) values of compounds 7a–g. The HOMO and LUMO energy values are in eV. These values provide information about the chemical stability and reactivity of the compounds, which correlate with their observed biological activities

Compound	HOMO (eV)	LUMO (eV)	Δ*E* (eV)
7a	–7.10	0.18	7.28
7b	–7.66	–0.83	6.83
7c	–7.60	–0.68	6.92
7d	–7.34	0.08	7.42
7e	–7.38	0.03	7.41
7f	–7.39	0.01	7.40
7g	–7.22	0.15	7.37

#### Binding affinity and interaction analysis

Molecular docking studies were conducted to explore the binding interactions of the synthesized pyrazolyl–thiazole derivatives (7a–g) with potential biological targets, particularly penicillin-binding proteins (PBPs) and sterol 14α-demethylase, to gain insights into their antimicrobial and antifungal activities.

**Fig. 3:**
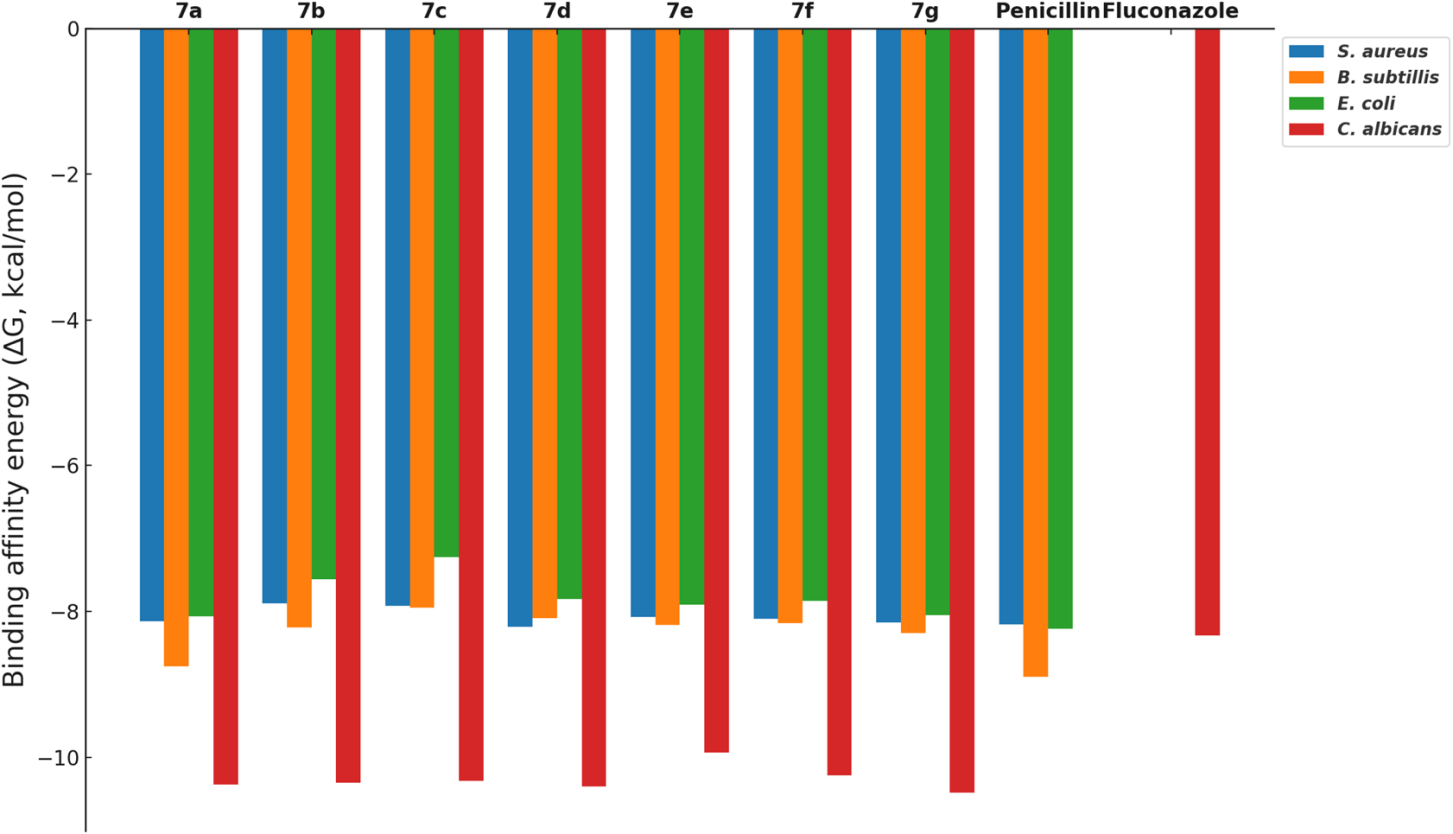
Binding affinity energies (Δ*G*, kcal mol^−1^) of compounds 7a–g when docked into the active sites of penicillin-binding proteins from *S. aureus* (PBP2a), *B. subtilis* (PBP4a), *E. coli* (PBP4), and sterol 14α-demethylase from *C. albicans*. Lower (more negative) Δ*G* values indicate stronger binding affinities, which are predictive of greater biological activity.

The docking simulations were performed using AutoDock Vina, where compounds 7a–g were docked into the active sites of the PBPs from Gram-positive (*S. aureus* and *B. subtilis*) and Gram-negative (*E. coli*) bacteria, as well as the fungal protein sterol 14α-demethylase from *C. albicans*. Penicillin and fluconazole were used as reference drugs in the docking studies. The binding affinities (Δ*G*, kcal mol^−1^) of the compounds were calculated, and the results, summarized in Fig. 3, reveal that the compounds generally show binding affinities comparable to or slightly better than those of the reference drugs.

Compounds 7d and 7g exhibited the best binding affinities among the synthesized derivatives, correlating with their observed superior inhibitory activities as reported in the experimental assays (Table 1 and Fig. 1). Specifically, compound 7g demonstrated strong binding to the active sites of both bacterial PBPs and the fungal sterol 14α-demethylase, suggesting its potential as a broad-spectrum antimicrobial agent.

#### Detailed interaction mechanisms and implications

**Fig. 4:**
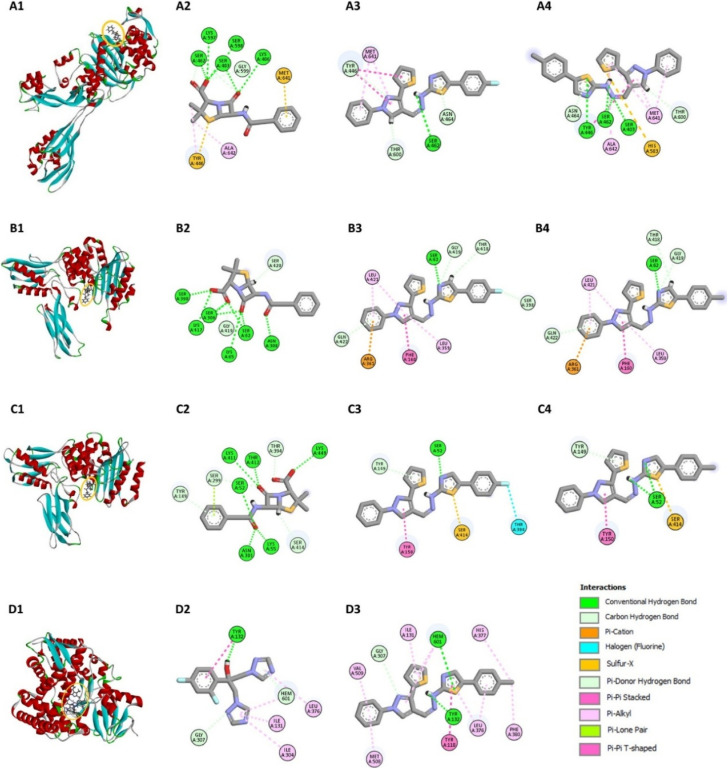
Molecular docking analysis of compounds 7d and 7g at target protein active sites. The binding modes and interactions of compounds 7d and 7g within the active sites of target proteins. A1–A4: Binding interactions with PBP2a of *S. aureus*. B1–B4: Binding interactions with PBP4 of *E. coli*. C1–C4: Binding interactions with PBP4a of *B. subtilis*. D1–D3: Binding interactions with sterol 14α-demethylase of *C. albicans*. The figures highlight specific amino acid residues involved in the binding interactions, such as hydrogen bonds, π–π stacking, and π–alkyl interactions.

The docking results provided detailed insights into the molecular interactions between the compounds and the target proteins. For example, according to Lim and Strynadka,^12^ the active site of PBP2a in *S. aureus* is formed by residues such as Ser403, Lys406, Tyr446, Ser462, and Met641, with Ser403 being the catalytic base with which inhibitors interact. Compound 7d formed hydrogen bonds with Ser462 and displayed π–π stacking interactions with Tyr446 and Met641, enhancing its binding affinity (Fig. 4A3) (ESI†). Similarly, compound 7g interacted with Ser403 and Tyr446 through hydrogen bonds, and with Met641 through π–π stacking interactions (Fig. 4A4), correlating with its observed antimicrobial activity against *S. aureus*.

For the penicillin-binding protein PBP4 of *E. coli*, Kishida *et al.*^13^ have identified key residues in the active site, including Ser62, Asn154, Asp155, Met156, Thr157, Gln158, and Phe160. Compound 7d demonstrated strong hydrogen bonding with Ser62 and π–π stacking with Phe160, contributing to its inhibitory activity (Fig. 4B3). Compound 7g showed similar interactions, with additional π–alkyl interactions with Leu421 and π–cation interactions with Arg361, further strengthening its binding affinity (Fig. 4B4). These interactions align with the experimental data, where 7g exhibited significant antimicrobial activity against *E. coli*.

In the PBP4a of *B. subtilis*, the active site includes residues such as Ser52, Lys55, Asp145, Tyr150, Ser299, and Asn301.^14^ Compound 7d formed hydrogen bonds with Ser52 and π–π stacking with Tyr150, while also interacting with Ser414 through a sulfur-x interaction, which enhances its binding to the protein (Fig. 4C3). Compound 7g exhibited similar binding patterns, including π-donor hydrogen bonds with Tyr149 and π–π stacking with Tyr150, supporting its high binding affinity and antimicrobial efficacy (Fig. 4C4).

For the antifungal target sterol 14α-demethylase of *C. albicans*, azoles that inhibit CYP51 activity typically interact with iron from the heme prosthetic group.^15^ The active site of CYP51 includes residues such as Glu116, Leu220, Tyr243, and Ile297.^15,16^ Compound 7g showed a higher binding affinity (Δ*G* = −10.49 kcal mol^−1^) compared to fluconazole (Δ*G* = −8.33 kcal mol^−1^), forming multiple interactions with key residues such as Tyr118, His377, and Met508, as well as with the heme group (Fig. 4D3). Although fluconazole's interaction with the heme iron occurs at a more favorable distance, 7g's overall interaction profile suggests it could serve as a template for further modifications to enhance its antifungal potency while potentially reducing toxicity. Detailed interaction shown in ESI (pages S14–S19†).

The docking studies suggest that the synthesized pyrazolyl-thiazole derivatives, particularly 7g, have promising potential as lead compounds for further drug development. Their ability to bind effectively to both bacterial and fungal targets, coupled with favourable binding affinities, underscores their potential as dual-action antimicrobial agents.

#### Absorption and emission spectra

The absorption and emission spectra of the compounds were calculated to predict their photophysical properties. The calculated absorption and emission wavelengths (*λ*_abs_ and *λ*_em_) are summarized in Table 5. All of the compounds absorb in the visible spectrum; however, only 7b (R = 4-NO_2_) and 7c (R = 3-NO_2_) emit in violet colour. Interestingly, a strong electron-donor group (7a) produces the largest Stokes shift (15 169 cm^−1^) followed by a weak electron-donor group (7g, 14 806 cm^−1^). This decreases progressively by using weak electron-withdrawing groups (7d (R = 4-F), 14 802 cm^−1^; 7e (R = 4-Cl), 14 390 cm^−1^; and 7f (R = 4-Br), 14 264 cm^−1^) and strong electron-withdrawing groups (7b (R = 4-NO_2_) 8372 cm^−1^; 7c (R = 3-NO_2_), 6386 cm^−1^).

**Table 5:** Calculated absorption (*λ*_abs_) and emission (*λ*_em_) wavelengths (in nm) of the compounds 7b–g. The data highlight the photophysical properties of these compounds, with a focus on Stokes shifts and the influence of different substituents on the phenyl ring

Compound	*λ*_abs_ (nm)	*λ*_em_ (nm)
7b	320	430
7c	315	425
7d	318	428
7e	317	426
7f	319	429
7g	316	427

### Structure–activity relationship (SAR) analysis

The SAR analysis of the compounds 7a–g reveals how different substituents on the phenyl ring specifically influence their antimicrobial and antioxidant activities. The observed biological activities can be linked directly to the electronic effects and steric properties introduced by these substituents.

The antimicrobial activity was found to be significantly influenced by the presence of electron-withdrawing groups such as nitro (NO_2_) and halogens (Cl, Br, F). Compounds with these substituents, particularly 7b (R = 4-NO_2_) and 7c (R = 3-NO_2_), exhibited substantial activity against *B. subtilis* and *B. megaterium*, with inhibition zones reaching up to 16 mm. The increased electrophilicity imparted by these groups likely enhances interactions with microbial targets by facilitating the formation of stronger bonds with enzyme active sites or cell membranes.

Moreover, the size and position of substituents on the phenyl ring played a crucial role in antimicrobial efficacy. Larger substituents or those at the *para* (4-) position, such as in 7d (R = 4-F), 7e (R = 4-Cl), and 7f (R = 4-Br), showed significant antimicrobial activity. This could be attributed to steric factors where the larger groups may enable better fitting into hydrophobic pockets of microbial enzymes, thereby enhancing binding interactions. The halogens (Cl, Br, F), being hydrophobic, further increased the overall hydrophobicity of the compounds, facilitating their penetration through microbial cell membranes. This property was particularly evident in the high activity of compounds 7d, 7e, and 7f against multiple strains.

For antioxidant activity, electron-donating groups such as methyl (CH_3_) significantly enhanced the radical scavenging abilities of the compounds. Compound 7g (R = 4-CH_3_) demonstrated notable DPPH and hydroxyl radical scavenging activities. The electron-donating nature of the methyl group stabilizes the radical form of the antioxidant by delocalizing the unpaired electron across the molecule, thus enhancing the compound's ability to neutralize free radicals. Additionally, the extended π-conjugation system present in the pyrazolyl–thiazole framework facilitates electron delocalization, further improving radical scavenging efficiency.

The antioxidant activity was also found to correlate well with the HOMO–LUMO energy gaps determined through computational studies. Compounds with larger HOMO–LUMO gaps, such as 7d and 7g, demonstrated better radical scavenging abilities, likely due to their increased electronic stability and reduced reactivity.

The dual antimicrobial and antioxidant activities of these pyrazolyl–thiazole derivatives suggest their potential as multifunctional therapeutic agents. The structural features contributing to both activities include substituents that modulate electron density and introduce steric effects, which can enhance both binding to microbial targets and radical stabilization. For example, the 4-nitro group in compound 7b and the 4-methyl group in compound 7g are associated with high activity in both antimicrobial and antioxidant assays.

Furthermore, the nearly coplanar arrangement of the pyrazolyl and thiazole rings, as revealed by the geometry optimization studies, facilitates π–π interactions, which are crucial for both antimicrobial binding and radical stabilization. The molecular docking results also support this observation, as compounds 7d and 7g formed strong π–π stacking interactions with key residues in the active sites of target proteins, correlating with their high experimental activities.

## Conclusions

In conclusion, the synthesized pyrazolyl–thiazole derivatives of thiophene demonstrated significant antimicrobial and antioxidant activities, making them promising candidates for further development as multifunctional therapeutic agents. The SAR analysis revealed that specific substituents on the phenyl ring significantly influence the biological activities of these compounds, with electron-withdrawing groups enhancing antimicrobial efficacy and electron-donating groups improving antioxidant properties. Computational studies provided valuable insights into the electronic properties and binding interactions of the compounds, further supporting the experimental results. The dual antimicrobial and antioxidant activities of these derivatives underscore their potential in combating drug-resistant pathogens and oxidative stress-related diseases. Future studies could incorporate solvent-phase calculations to explore the influence of solvent effects on the electronic and structural properties of the compounds.

## Experimental section

### Materials and methods

All reagents and solvents were purchased from commercial suppliers and used without further purification unless otherwise noted. Analytical thin-layer chromatography (TLC) was performed on silica gel plates, and the compounds were visualized using UV light. Column chromatography was carried out using silica gel (60–120 mesh). Melting points were determined using a capillary melting point apparatus and are uncorrected.

### Synthesis of pyrazolyl–thiazole derivatives

The synthetic route for the pyrazolyl–thiazole derivatives of thiophene 7b–g is outlined in Scheme 1.

#### Synthesis of hydrazone intermediate

Acetyl thiophene 1 (1.12 g, 1 equiv., 0.01 mol) was added to phenyl hydrazine 2 (0.99 mL, 1 equiv., 0.01 mol) in the presence of conc. H_2_SO_4_ (0.5 mL). The mixture was stirred at room temperature for 2 hours. The resulting hydrazone intermediate, 1-phenyl-2-(1-(thiophen-2-yl)ethylidene)hydrazine (2.07 g, 96% yield), was collected by filtration, washed with ethanol, and purified by recrystallization.

##### Recrystallization

The crude product was dissolved in hot ethanol (approximately 20 mL per gram of product) and allowed to cool to room temperature. The solution was further chilled in an ice bath to promote crystallization. The resulting crystals were filtered, washed with cold ethanol, and dried under vacuum to obtain the purified product.

#### Synthesis of pyrazole-4-carbaldehyde

The hydrazone intermediate was cyclized using POCl_3_ (4.65 mL, 0.05 mol) in DMF (10 mL) to form pyrazole-4-carbaldehyde 3, 1-phenyl-3-(thiophen-2-yl)-1*H*-pyrazole-4-carbaldehyde. The reaction mixture was heated under reflux for 4 hours, then cooled to room temperature. The solid product (2.09 g, 82% yield) was filtered, washed with water, and recrystallized from ethanol.^10^

##### Recrystallization

The crude product was dissolved in a minimum amount of hot ethanol, and petroleum ether was added slowly to the solution. The mixture was then kept at 0 °C to promote crystallization. The resulting crystals were filtered, washed with cold petroleum ether, and dried under vacuum to obtain the purified product.

#### Synthesis of thiosemicarbazone derivative

Pyrazole-4-carbaldehyde 3 (2.09 g, 0.01 mol) was reacted with thiosemicarbazide 4 (0.91 g, 0.01 mol) in ethanol (10 mL) with acetic acid (0.5 mL) as a catalyst. The mixture was refluxed for 1 hour. Upon completion, the reaction mixture was cooled to room temperature, and the solid product 5, 2-((1-phenyl-3-(thiophen-2-yl)-1*H*-pyrazol-4-yl)methylene)hydrazine-1-carbothioamide (2.78 g, 85% yield), was filtered, washed with ethanol, and recrystallised using ethanol and petroleum ether.

#### Synthesis of pyrazolyl-thiazole derivatives

The thiosemicarbazone intermediate 5 (3.27 g, 1 equiv., 0.01 mol) was reacted with various substituted phenacyl bromides 6a–g (1 equiv., 0.01 mol) in ethanol (10 mL) under reflux conditions for 3 hours. The reaction mixtures were then cooled to room temperature, and the solid products were filtered, washed with ethanol, and recrystallized recrystallised using ethanol and petroleum ether to obtain the pure pyrazolyl-thiazole derivatives 7b–g.

### Characterization of synthesized compounds

The structures of the synthesized compounds were confirmed by NMR spectroscopy and mass spectrometry. ^1^H and ^13^C NMR spectra were recorded on a 400 MHz NMR spectrometer using CDCl_3_ or DMSO-d_6_ as solvents. Chemical shifts (*δ*) are reported in parts per million (ppm) relative to the internal standard tetramethylsilane (TMS). The peak assignment for NMR spectra was done and mentioned in the characterization for each compound. Mass spectra were recorded on an ESI-MS spectrometer.

#### 2-(5-(4-Methoxyphenyl)thiazole-2-yl)-1-((1-phenyl-3-(thiophen-2-yl)-1*H*-pyrazol-4-yl)methylene)hydrazine (7a)

Phenyl-substituted 2-bromo-1-(4-methoxyphenyl)ethan-1-one (2.291 g, 0.01 mol) was added to the thiosemicarbazone derivative in ethanol and refluxed for 3 hours. Yield: 3.661 g, 80%; mp: 235–237 °C; ^1^H NMR (400 MHz, DMSO-d_6_): *δ* 8.92 (s, 1H pyrazolyl-H), 7.82 (s, 1H, thiazolyl-H), 8.35 (s, 1H, HC=N),7.2–7.6 (m, 5H, phenyl ring), 7.94 (d, 2H, Ar-H), 7.74 (d, 2H, Ar-H), 6.95–7.15 (m, 3H, thiophene), 3.76 (s, 3H, OCH_3_); ^13^C NMR (100 MHz, DMSO-d_6_): *δ* 55, 102, 114, 116, 119, 127, 128, 129, 130, 134, 139, 145, 159, 168; MS (EI, 70 eV): *m*/*z* (%): 458 (M + H, 100).

#### 2-(5-(4-Nitrophenyl)thiazole-2-yl)-1-((1-phenyl-3-(thiophen-2-yl)-1*H*-pyrazol-4-yl)methylene)hydrazine (7b)

Phenyl-substituted 2-bromo-1-(4-nitrophenyl)ethan-1-one (2.44 g, 0.01 mol) was added to the thiosemicarbazone derivative in ethanol and refluxed for 3 hours. Yield: 4.347 g, 92%; mp: 194–196 °C; ^1^H NMR (400 MHz, DMSO-d_6_): *δ* 12.18 (bs, 1H, N–H), 8.91 (s, 1H, pyrazolyl-H), 7.7 (s, 1H, thiazolyl-H), 8.31 (s, 1H, HC=N), 7.5–7.9 (m, 5H, phenyl ring), 8.29 (d, 2H, Ar-H), 7.96 (d, 2H, Ar-H), 7.24–7.40 (m, 3H, thiophene); ^13^C NMR (100 MHz, DMSO-d_6_): *δ* 108, 116, 119, 124, 126, 127, 128, 129, 130, 134, 139, 145, 146, 168; MS (EI, 70 eV): *m*/*z* (%): 473 (M + H, 100).

#### 2-(5-(3-Nitrophenyl)thiazole-2-yl)-1-((1-phenyl-3-(thiophen-2-yl)-1*H*-pyrazol-4-yl)methylene)hydrazine (7c)

Phenyl-substituted 2-bromo-1-(3-nitrophenyl)ethan-1-one (2.44 g, 0.01 mol) was added to the thiosemicarbazone derivative in ethanol and refluxed for 3 hours. Yield: 4.347 g, 92%; mp: 125–127 °C; ^1^H NMR (400 MHz, DMSO-d_6_): *δ* 8.94 (s, 1H pyrazolyl-H), 8.33 (s, 1H, thiazolyl-H), 8.69 (s,1H, HC=N), 8.3–7.9 (m, 4H, Ar-H), 7.58–7.85 (m, 5H, phenyl ring), 7.25–7.56 (m, 3H, thiophene); ^13^C NMR (100 MHz, DMSO-d_6_): *δ* 104, 115, 116, 119, 127, 128, 129, 130, 134, 139, 145, 168; MS (EI, 70 eV): *m*/*z* (%): 473 (M + H, 100).

#### 2-(5-(4-Fluorophenyl)thiazole-2-yl)-1-((1-phenyl-3-(thiophen-2-yl)-1*H*-pyrazol-4-yl)methylene)hydrazine (7d)

Phenyl-substituted 2-bromo-1-(4-fluorophenyl)ethan-1-one (2.17 g, 0.01 mol) was added to the thiosemicarbazone derivative in ethanol and refluxed for 3 hours. Yield: 3.965 g, 89%; mp: 240–242 °C; ^1^H NMR (400 MHz, DMSO-d_6_): *δ* 11.38 (bs, 1H, N–H), 9.18 (s, 1H, pyrazolyl-H), 8.68 (s, 1H, thiazolyl-H), 8.31 (s, 1H, HC=N), 7.4–7.8 (m, 5H, phenyl ring), 8.39 (d, 2H, Ar-H), 7.94 (d, 2H, Ar-H), 7.23–7.40 (m, 3H, thiophene); ^13^C NMR (100 MHz, DMSO-d_6_): *δ* 106, 116, 117, 118, 119, 120, 122, 125, 126, 127, 128, 129, 130, 132, 134, 135, 136, 139, 148, 168; MS (EI, 70 eV): *m*/*z* (%): 446 (M + H, 100).

#### 2-(5-(4-Chlorophenyl)thiazole-2-yl)-1-((1-phenyl-3-(thiophen-2-yl)-1*H*-pyrazol-4-yl)methylene)hydrazine (7e)

Phenyl-substituted 2-bromo-1-(4-chlorophenyl)ethan-1-one (2.335 g, 0.01 mol) was added to the thiosemicarbazone derivative in ethanol and refluxed for 3 hours. Yield: 3.596 g, 78%; mp: 154–156 °C; ^1^H NMR (400 MHz, DMSO-d_6_): *δ* 8.93 (s, 1H pyrazolyl-H), 7.85 (s, 1H, thiazolyl-H), 8.31 (s, 1H, HC=N), 7.4–7.6 (m, 5H, phenyl ring), 7.97 (d, 2H, Ar-H), 7.88 (d, 2H, Ar-H), 7.24–7.41 (m, 3H, thiophene); ^13^C NMR (100 MHz, DMSO-d_6_): *δ* 104, 116, 119, 127, 128, 129, 130, 134, 135, 145, 168; MS (EI, 70 eV): *m*/*z* (%): 462 (M + H, 100).

#### 2-(5-(4-Bromophenyl)thiazole-2-yl)-1-((1-phenyl-3-(thiophen-2-yl)-1*H*-pyrazol-4-yl)methylene)hydrazine (7f)

Phenyl-substituted 2-bromo-1-(4-bromophenyl)ethan-1-one (2.779 g, 0.01 mol) was added to the thiosemicarbazone derivative in ethanol and refluxed for 3 hours. Yield: 3.798 g, 75%; mp: 180–182 °C; ^1^H NMR (400 MHz, DMSO-d_6_): *δ* 8.89 (s, 1H pyrazolyl-H), 7.76 (s, 1H, thiazolyl-H), 8.26 (s, 1H, HC=N), 7.5–7.7 (m, 5H, phenyl ring), 7.90 (d, 2H, Ar-H), 7.72 (d, 2H, Ar-H), 7.09–7.48 (m, 3H, thiophene); ^13^C NMR (100 MHz, DMSO-d_6_): *δ* 116, 118, 121, 126, 127, 129, 130, 131, 138, 145, 167; MS (EI, 70 eV): *m*/*z* (%): 506 (M + H, 100).

#### 2-(5-(4-Methylphenyl)thiazole-2-yl)-1-((1-phenyl-3-(thiophen-2-yl)-1*H*-pyrazol-4-yl)methylene)hydrazine (7g)

Phenyl-substituted 2-bromo-1-(4-methylphenyl)ethan-1-one (2.131 g, 0.01 mol) was added to the thiosemicarbazone derivative in ethanol and refluxed for 3 hours. Yield: 3.798 g, 86%; mp: 190–192 °C; ^1^H NMR (400 MHz, DMSO-d_6_): *δ* 8.92 (s, 1H pyrazolyl-H), 7.86 (s, 1H, thiazolyl-H), 8.30 (s, 1H, HC=N), 7.38–7.67 (m, 5H, phenyl ring), 7.97 (d, 2H, Ar-H), 7.75 (d, 2H, Ar-H), 7.21–7.25 (m, 3H, thiophene), 2.27 (s, 3H, CH_3_); ^13^C NMR (100 MHz, DMSO-d_6_): *δ* 103, 116, 119, 126, 127, 128, 129, 130, 134, 137, 138, 139, 145, 147, 168; MS (EI, 70 eV): *m*/*z* (%): 442 (M + H, 100).

### Antimicrobial activity

The antimicrobial activities of the synthesized pyrazolyl–thiazole derivatives 7b–g were evaluated against a panel of bacterial and fungal strains. The bacterial strains tested included *E. coli*, *B. subtilis*, *B. megaterium*, and *S. aureus*. The fungal strains tested included *A. niger*, *A. oryzae*, *Rhizopus*, and *C. albicans*.

#### Disk diffusion method

The antimicrobial activity was assessed using the Kirby–Bauer disk diffusion method. Stock solutions of the synthesized compounds were prepared at 1 mg mL^−1^ in dimethyl sulfoxide (DMSO) and diluted to a final concentration of 10 μg per disc. Sterile disks were impregnated with 10 μL of the compound solutions and placed on agar plates inoculated with a standardized microbial suspension (approximately 1 × 10^8^ CFU mL^−1^). The plates were incubated at 37 °C for 24 hours. Zones of inhibition around each disk were measured in millimeters to determine the antimicrobial effectiveness of the compounds. Amoxicillin (10 μg) was used as a positive control for bacterial strains, and fluconazole (10 μg) was used for fungal strains, while DMSO served as the negative control.^17^

#### Minimum inhibitory concentration (MIC)

The MIC values of the synthesized compounds were determined using the resazurin microtiter assay (REMA). Serial dilutions of the compounds were prepared in 96-well plates, ranging from 0.5 to 128 μg mL^−1^. Each well was inoculated with 100 μL of microbial suspension at a density of 1 × 10^6^ CFU mL^−1^. Plates were incubated at 37 °C for 24 hours. The MIC values were determined as the lowest concentration of the compounds that completely inhibited visible microbial growth. All assays were performed in triplicate, with amoxicillin and fluconazole as positive controls and DMSO as the negative control.^18^

### Antioxidant activity

The antioxidant activities of the synthesized pyrazolyl–thiazole derivatives 7b–g were evaluated using the DPPH (2,2-diphenyl-1-picrylhydrazyl) radical scavenging assay and the hydroxyl radical scavenging assay.^19^

#### DPPH radical scavenging assay

The DPPH radical scavenging activity was measured by mixing a solution of DPPH (0.1 mM) in methanol with various concentrations of the synthesized compounds (10–100 μg mL^−1^). The mixture was incubated at room temperature in the dark for 30 minutes, and the absorbance was measured at 517 nm using a UV-vis spectrophotometer. Ascorbic acid was used as a positive control.^19^ The percent radical scavenging activity was calculated using the formula:
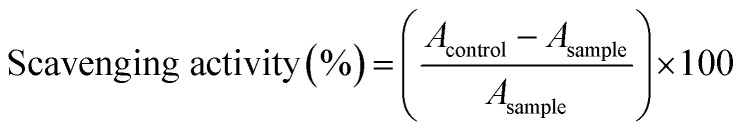


#### Hydroxyl radical scavenging assay

The hydroxyl radical scavenging activity was assessed by generating hydroxyl radicals through the Fenton reaction and measuring the degradation of deoxyribose. The reaction mixture contained deoxyribose (2.8 mM), FeSO_4_ (0.1 mM), EDTA (0.1 mM), H_2_O_2_ (1 mM), and various concentrations of the synthesized compounds (10–100 μg mL^−1^). The mixture was incubated at 37 °C for 1 hour, and the thiobarbituric acid-reactive substances (TBARS) formed were measured at 532 nm using a spectrophotometer. Mannitol was used as a positive control for this assay.

### Computational studies

Gaussian 09 software was used to optimize the geometries and compute the electronic properties of the synthesized compounds 7b–g.^20^ Geometry optimizations at gas-phase with no constraints were done using the long-range corrected ωB97X-D hybrid functional along with Ahlrichs' def2-TZVPP basis set. All geometry optimizations were performed in the gas phase to focus on intrinsic molecular properties and reduce computational complexity. This approach is commonly employed in similar studies and provides reliable insights into electronic and structural properties.^21^ The absence of imaginary frequencies in the vibrational analysis confirmed that the optimized structures were true minima on the potential energy surface. Key computational results, including HOMO–LUMO energy values, thermodynamic properties, and absorption/emission spectra, are detailed in the results section and ESI.†

#### Molecular docking studies

The 2D structures of the synthesized compounds were drawn with ChemSketch ACD/Labs and the protonation states were calculated at pH 7.40 with MarvinSketch Chemaxon. The protein used in this study were obtained from the Protein Data Bank. Penicillin-binding proteins: PBP2 from *S. aureus* (PDB 1MWT), PBP4 from *E. coli* (PDB 2EX8), PBP4a from *B. subtillis* (PDB 1W5D). Sterol 14α-demethylase or CYP51 from *C. albicans* (PDB 5TZ1).

The molecular docking studies were carried out in the Molegro Virtual Docker software. The validation of the molecular docking method was performed by re-docking 12 methods based on the combination of 4 scoring functions and 3 search algorithms (data show in ESI†). In each method, 10 runs, a maximum of 1500 iterations and a population of 50 poses, a search sphere of 10 Å and a grid of 0.20 Å were performed. The method was chose using an RMSD value <2.0 Å as a criterion.^22^ The binding energy was obtained in the PROtein binDIng enerGY prediction (PRODIGY) server and Discovery Studio BIOVIA was used to visualize the ligand–receptor interactions.

## Data availability

All data supporting the findings of this study are available within the article and its ESI† files. Additional datasets generated and analyzed during the current study, including raw NMR spectra, mass spectrometry data, and detailed computational outputs, are available from the corresponding author upon reasonable request.

## Author contributions

Conceptualization, S. V. P.; methodology, S. K. B., S. A. K., A. A. P., R. A. M., G. M., and S. V. P.; software, T. J. P., J. O. C. J. H, J. A. A. S., and J. L. O. R.; validation, S. K. B., T. J. P., R. A. M., J. L. O. R., and S. V. P.; formal analysis, S. K. B, S. V. P.; investigation, S. K. B., T. J. P., S. A. K., A. A. P., R. A. M. E. D. A., and S. V. P.; resources, S. V. P., G. M. and E. D. A.; data curation, T. J. P. and S. V. P.; writing—original draft preparation, T. J. P., R. A. M., J. O. C. J. H., J. A. A. S.; writing—review and editing, T. J. P., J. O. C. J. H., J. L. O. R. and S. V. P.; visualization, E. D. A. and S. V. P.; supervision, R. A. M. and S. V. P.; project administration, S. V. P.; funding acquisition, G. M., E. D. A. and S. V. P. All authors have read and agreed to the published version of the manuscript.

## Conflicts of interest

There are no conflicts to declare.

## Supplementary Material

RA-014-D4RA06228K-s001
