# Supplementary material for: Synthesis, characterization, biological activities, and computational studies of pyrazolyl–thiazole derivatives of thiophene
Source: RSC Adv. 2024 Dec 10;14(52):39004–16. doi: 10.1039/d4ra06228k (PMC11629146; doi:10.1039/d4ra06228k)
Supplement: RA-014-D4RA06228K-s001 [file RA-014-D4RA06228K-s001.pdf]

Supplementary Information (SI) for RSC Advances.
This journal is © The Royal Society of Chemistry 2024

Supporting Information

Synthesis, Characterization, Biological Activities and Computational Studies of Pyrazolyl-Thiazole Derivatives of Thiophene

Seema K. Bhagwat,[a] Tushar Janardan Pawar,[b] Sayali A. Kulkarni,[a] Amar A. Patil,[a] Rahul Ashokrao More,[c] J. Oscar C. Jimenez-Halla,[d] Juan Andres Alvarado-Salazar,[e] Jose Luis Olivares-Romero,[b] Ghazala Muteeb,*[f] Enrique Delgado-Alvarado,*[g] and Sachin V. Patil*[a]

a. *Department of Chemistry, Research Centre HPT Arts and RYK Science College (Affiliated to S. P. Pune University), Nashik, 422005, Maharashtra, India.*
b. *Red de Estudios Moleculares Avanzados, Campus III, Instituto de Ecología, A. C., Carretera Antigua a Coatepec 351, 91073, Xalapa, Veracruz, México.*
c. *Department of Microbiology, Dayanand Science College, Latur, 413512, Maharashtra, India.*
d. *Departamento de Química, División de Ciencias Naturales y Exactas, Universidad de Guanajuato, Noria Alta S/N, 36050, Guanajuato, México.*
e. *Carrera de Química-Farmacéutico-Biológica, Facultad de Estudios Superiores Zaragoza, UNAM, 09230 Ciudad de México, México.*
f. *Department of Nursing, College of Applied Medical Sciences, King Faisal University, Al-Ahsa, Saudi Arabia.*
g. *Micro and Nanotechnology Research Center, Universidad Veracruzana, Blvd. Av. Ruiz Cortines No. 455 Fracc. Costa Verde, Boca del Río, Veracruz 94294, México.*

Table of Content

1. Biological Activity S2
2. Computational Methodology S4
3. Molecular Docking S14
4. NMR Spectra S22
5. References S35

1. Biological Activity

Disk diffusion assay

The antituberculosis potential of synthesized compounds was evaluated using **Kirby-Bauer disk diffusion** method.[1,3] Briefly, each sterile disk (Himedia Pvt. Ltd. Mumbai) loaded with synthesized compounds 50 µL (1 mg/ mL) and dried. Each disk was then placed on the surface of the sterile solidified Middlebrook 7H9 agar medium which was spreaded with 24 hrs old inoculums *M. Tuberculosis* $H_{37}Ra$ and Rifampicin (1mg/ mL). The plates were kept in refrigerator for diffusion for 1 hr and then transferred to the incubator at 37ºC for 24-48 hrs. After incubation, the zones around the discs were measured by the zone scale (Himedia Pvt. Ltd. Mumbai).

Resazurin microtiter assay (REMA) for calculating MIC

The REMA plate assay was carried out as described elsewhere.[2,3] Briefly, 100 µL of Middlebrook 7H9 broth medium was dispensed in each well of a sterile flat-bottom 96-well plate, and serial twofold dilutions of each synthesized compounds were prepared directly in the plate. Fifty micro litres of inoculums was added to each well. Sterile cold water was added to all perimeter wells to avoid evaporation during the incubation. The plate was covered, sealed with the sterile plastic bag, and incubated at 37°C for 24 hrs. After 24 hrs of incubation, 30 µL of resazurin solution (0.01% in sterile deionized water) was added to each well, and the plate was re-incubated for 12 hrs. A change in color from blue to pink indicated the growth of bacteria, and the MIC was defined as the lowest concentration of drug that prevented this change in color. The drug concentration ranges used were as follows: for synthesized compounds and standards 0.97- 500 µg/mL.

DPPH (2, 2-diphenyl-1-picrylhydrazyl radical scavenging assay)

Free radical damage is one of the important aspect in the tuberculosis management. DPPH is stable reagent used in this spectrophotometric assay.[4] Briefly, the assay was performed by mixing of equal quantity of DPPH solution and the test compound, so that the final volume is made up to 3 mL incubate the samples for 20 min, read the absorbance at 517 nm using (Shimadzu UV Vis- Spectrophotometer). Ascorbic acid (1 mM) was used as a standard. Percent inhibition was calculated using following formula:

% radical scavenging activity = 1 – T/C x 100

OH (Hydroxyl radical assay)

The OH radical scavenging activity was demonstrated with Fenton reaction.[3,4] Briefly, the typical reaction mixture contained 60 µL of $FeCl_2$ (1 mM), 90 µL of 1-10 phenanthroline (1 mM), and 2.4 mL of phosphate buffer (0.2 M, pH 7.8), 150 µL of H_2O_2 (0.17 M) and 1.5 mL Of individual synthesized compound (1 mg/mL). The reaction was started by adding H_2O_2. After 5 min incubation at room temperature, the absorbance was read at 560 nm. Ascorbic acid (1 mM) was used as reference.

% radical scavenging activity = 1 – T/C x 100

2. Computational Methodology

All electronic structure calculations were performed using the Gaussian 16 software package.[4] Geometry optimizations for all compounds were performed in the gas phase using the B3LYP functional with the 6-31G(d) basis set. Harmonic frequency calculations confirmed the absence of imaginary frequencies, ensuring that all optimized structures correspond to local minima on the potential energy surface.

Subsequent single-point energy calculations were carried out using the ωB97X-D[5] functional with the Ahlrichs def2-TZVPP[6] basis set for all atoms (H, C, N, O, S, F, Cl, and Br). Thermal corrections for Gibbs free energy were calculated from thermochemistry analyses considering an ideal gas at 298.15 K and 1 atm.

Gas-phase calculations for absorption and emission spectra of all compounds were performed using the τ-HCTHhyb[7] density functional for the ground and first excited (singlet) states, respectively. Key computational results, including optimized geometries and energy values, are provided in Table S1 and Figure S1.

Geometry optimizations were carried out in the gas phase to focus on the intrinsic molecular properties and reduce computational demands. These results are expected to be reliable for the compounds studied, based on comparisons with similar systems in the literature.[8]

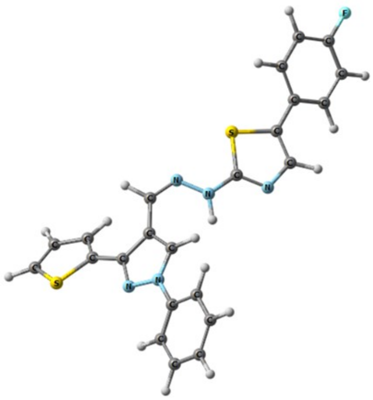

4
(0.0)

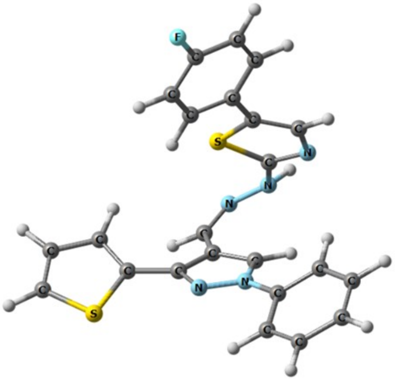

4_{cis}
(5.3)

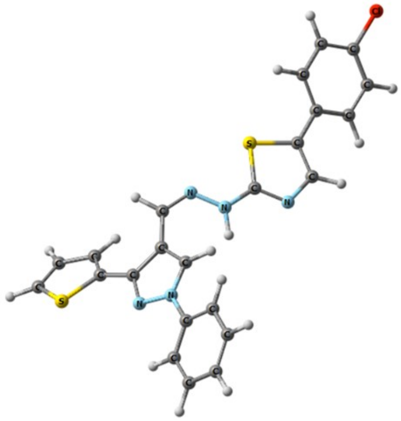

5
(0.0)

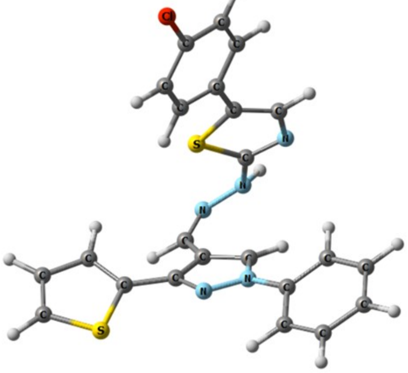

5_{cis}
(5.1)

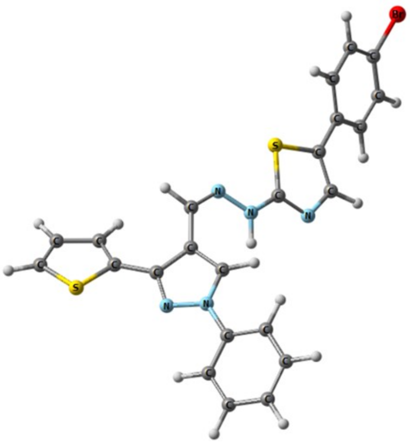

6
(0.0)

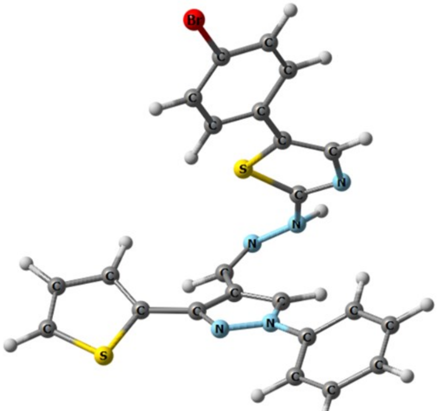

6_{cis}
(5.5)

Figure S1. Conformational study of the species **1-6**. Energies shown are in Gibbs free energies (kcal/mol).

Table S1. Cartesian coordinates (x-y-z format) of the calculated geometry optimizations performed at the ωB97X-D/def2tzvpp level.

**1**				$\mathbf{1_{cis}}$			
E(scf) = -2161.33133142 a.u.				E(scf) = -2161.32289107 a.u.			
C	4.025189	1.081779	-0.106858	C	2.718674	0.763354	-0.226987
C	2.763712	0.545601	-0.516755	C	2.529996	0.306252	1.114242
C	2.996078	-0.820073	-0.586164	C	2.304371	-1.054487	0.970118
N	4.293525	-1.023085	-0.253696	N	2.372884	-1.329479	-0.349309
H	2.340661	-1.641214	-0.868648	H	2.133721	-1.826107	1.716477
N	4.919578	0.118902	0.047071	N	2.621232	-0.238779	-1.086082
C	4.985467	-2.261679	-0.166207	C	2.189594	-2.591698	-0.975550
C	4.556496	-3.364141	-0.907753	C	1.397582	-3.566592	-0.365782
C	6.098068	-2.359444	0.671811	C	2.803191	-2.837796	-2.204859
C	5.234117	-4.576296	-0.789941	C	1.241223	-4.805893	-0.983757
H	3.710514	-3.281433	-1.594194	H	0.883768	-3.357421	0.575939
C	6.772951	-3.573013	0.771634	C	2.628894	-4.075979	-2.817128
H	6.421006	-1.480310	1.231281	H	3.403165	-2.052602	-2.667537
C	6.342383	-4.686100	0.048682	C	1.854881	-5.065086	-2.208702
H	4.896188	-5.438206	-1.371659	H	0.619904	-5.568541	-0.506542
H	7.643566	-3.648710	1.428896	H	3.108273	-4.269543	-3.780624
H	6.872894	-5.637976	0.135377	H	1.723730	-6.036195	-2.693411
C	4.377605	2.477610	0.133351	C	2.958110	2.127533	-0.688488
C	3.556840	3.532419	0.460627	C	2.546538	3.311026	-0.119749
S	6.031239	2.982407	0.039039	S	3.836108	2.414322	-2.152349
C	4.269583	4.754041	0.629205	C	2.950832	4.453091	-0.868655
H	2.479873	3.431686	0.608359	H	1.941705	3.361870	0.788088
C	5.615480	4.601885	0.430030	C	3.657258	4.114849	-1.991484
H	3.800195	5.703341	0.893576	H	2.718820	5.482253	-0.588393
H	6.391049	5.365511	0.497104	H	4.080083	4.784450	-2.741229
C	1.564472	1.300034	-0.889433	C	2.654971	1.091024	2.336557
H	1.714732	2.262301	-1.392184	H	3.289319	1.984434	2.275395
N	0.338831	0.975643	-0.707114	N	2.178149	0.877640	3.508276
N	0.039749	-0.164278	-0.060427	N	1.331393	-0.198614	3.685480
H	0.733553	-0.660262	0.500618	H	1.303220	-0.493713	4.657117
C	-1.273690	-0.449522	0.205575	C	0.107031	-0.353654	3.073162
S	-2.549749	0.521390	-0.464141	S	-0.503855	0.711051	1.833643
C	-3.004664	-1.500251	1.017038	C	-1.873018	-1.229793	2.728128
C	-3.698057	-0.537142	0.331741	C	-1.979342	-0.221741	1.805467
H	-3.471400	-2.306455	1.587792	H	-2.642980	-1.985909	2.899966
N	-1.642884	-1.451259	0.945337	N	-0.708978	-1.306013	3.429703
C	-5.146165	-0.349365	0.217218	C	-3.066017	0.065622	0.865710
C	-5.706107	0.367353	-0.855180	C	-2.821561	0.727242	-0.350612
C	-6.015015	-0.891422	1.182553	C	-4.382573	-0.332586	1.160019
C	-7.080081	0.535832	-0.968690	C	-3.850806	0.979687	-1.248445
H	-5.055222	0.791618	-1.625001	H	-1.804289	1.035423	-0.609204

C	-7.390502	-0.739580	1.074615	C	-5.417692	-0.095478	0.265524
H	-5.606235	-1.424287	2.044209	H	-4.601926	-0.819997	2.113056
C	-7.908104	-0.024864	-0.002079	C	-5.138345	0.560520	-0.929867
H	-7.519014	1.088376	-1.800215	H	-3.667905	1.487968	-2.195885
H	-8.069621	-1.155393	1.819977	H	-6.441893	-0.399487	0.485200
N	-9.363646	0.146032	-0.117274	N	-6.232165	0.819329	-1.878249
O	-9.780332	0.783452	-1.060092	O	-5.957525	1.400482	-2.905420
O	-10.057041	-0.361578	0.737463	O	-7.340366	0.434774	-1.573456
**2**				$\mathbf{2_{cis}}$			
E(scf) = -2161.33027589 a.u.				E(scf) = -2161.32135090 a.u.			
C	3.979887	-1.011411	0.008407	C	-2.212788	-0.419078	-1.013165
C	2.735125	-0.550682	0.542561	C	-2.342378	0.983768	-1.255041
C	2.911472	0.821173	0.645336	C	-1.087256	1.357423	-1.682984
N	4.165724	1.097061	0.213768	N	-0.296915	0.251769	-1.655554
H	2.246273	1.600471	1.011329	H	-0.718652	2.337032	-1.973764
N	4.814431	-0.002425	-0.182219	N	-0.977739	-0.827380	-1.254968
C	4.790487	2.369913	0.114455	C	1.102152	0.166329	-1.874490
C	4.389677	3.419459	0.943290	C	1.877924	1.324882	-1.979742
C	5.808057	2.554956	-0.823622	C	1.707233	-1.091051	-1.939092
C	4.998828	4.666428	0.814335	C	3.257633	1.218565	-2.145190
H	3.619985	3.268103	1.703874	H	1.420851	2.313868	-1.912847
C	6.415808	3.802560	-0.934751	C	3.087164	-1.182098	-2.102348
H	6.111364	1.715937	-1.451651	H	1.087694	-1.983001	-1.842748
C	6.011623	4.863322	-0.123240	C	3.869858	-0.032065	-2.206111
H	4.683673	5.486439	1.465281	H	3.860755	2.127879	-2.212769
H	7.211248	3.946953	-1.671036	H	3.558362	-2.168037	-2.140978
H	6.489507	5.841906	-0.218441	H	4.954167	-0.110714	-2.319021
C	4.370800	-2.380672	-0.312857	C	-3.198538	-1.347929	-0.473659
C	3.571080	-3.454473	-0.630190	C	-4.500303	-1.114890	-0.098700
S	6.044925	-2.821379	-0.344996	S	-2.750127	-2.996282	-0.171893
C	4.317073	-4.638616	-0.894691	C	-5.140020	-2.274838	0.427469
H	2.483605	-3.391401	-0.704431	H	-4.985391	-0.141450	-0.187056
C	5.666329	-4.440251	-0.775225	C	-4.311661	-3.363279	0.447599
H	3.867570	-5.594847	-1.168519	H	-6.173785	-2.294972	0.777739
H	6.463891	-5.169099	-0.923407	H	-4.540375	-4.371802	0.793652
C	1.606565	-1.376467	0.979397	C	-3.528251	1.852513	-1.062584
H	1.842115	-2.348863	1.426752	H	-4.338080	1.745201	-1.797732
N	0.356087	-1.107640	0.911123	N	-3.784929	2.733069	-0.168311
N	-0.052113	0.041110	0.346296	N	-2.996926	3.042329	0.888773
H	0.567463	0.598320	-0.243402	H	-3.229735	3.954147	1.264461
C	-1.396969	0.254463	0.180507	C	-1.684768	2.610137	1.093732
S	-2.563378	-0.831601	0.874673	S	-1.383765	1.036944	1.766041
C	-3.241093	1.227798	-0.461707	C	0.500091	2.530182	0.986594
C	-3.824594	0.188559	0.211846	C	0.303315	1.248527	1.449586
H	-3.794401	2.026802	-0.960985	H	1.472011	2.948421	0.718275

N	-1.875621	1.264176	-0.478639	N	-0.616868	3.280472	0.781547
C	-5.250353	-0.102454	0.399869	C	1.269455	0.143288	1.513299
C	-5.708588	-0.892636	1.467494	C	2.628756	0.386944	1.282334
C	-6.198569	0.415195	-0.494295	C	0.849065	-1.184819	1.683216
C	-7.065435	-1.155943	1.642794	C	3.507953	-0.680857	1.201887
H	-4.989104	-1.300962	2.183696	H	3.017923	1.393123	1.130718
C	-7.545510	0.149960	-0.291639	C	1.757681	-2.239724	1.632900
H	-5.903850	1.011956	-1.358056	H	-0.212102	-1.407435	1.826057
C	-8.007315	-0.631889	0.762099	C	3.103803	-2.000224	1.379293
H	-7.392926	-1.774197	2.482587	H	1.401606	-3.264008	1.766979
H	-9.075689	-0.817178	0.875099	H	3.832379	-2.807405	1.301549
O	-8.098315	1.389286	-2.152861	N	4.913644	-0.404329	0.861241
N	-8.524197	0.709213	-1.245661	O	5.631889	-1.354849	0.640754
O	-9.693180	0.454105	-1.059293	O	5.262619	0.754451	0.802148
**3**				$\mathbf{3_{cis}}$			
E(scf) = -2071.34235740 a.u.				E(scf) = -2071.33421540 a.u.			
C	-3.831755	-1.036138	0.074281	C	2.531472	0.732135	-0.219701
C	-2.546629	-0.540561	0.462996	C	2.351729	0.324376	1.138307
C	-2.726536	0.833818	0.502958	C	2.109881	-1.037028	1.043093
N	-4.015832	1.080181	0.165611	N	2.160921	-1.358594	-0.267693
H	-2.054444	1.629670	0.817383	H	1.934797	-1.780112	1.816576
N	-4.690636	-0.043461	-0.095985	N	2.414836	-0.297493	-1.043850
C	-4.662879	2.341479	0.085471	C	1.949127	-2.638020	-0.844931
C	-3.920273	3.499047	-0.156415	C	1.147598	-3.575741	-0.190156
C	-6.047746	2.408967	0.252238	C	2.544871	-2.940121	-2.070502
C	-4.567845	4.731831	-0.208732	C	0.965249	-4.834208	-0.760872
H	-2.842295	3.444506	-0.326045	H	0.647718	-3.320932	0.748214
C	-6.683800	3.645462	0.186336	C	2.344577	-4.197088	-2.634464
H	-6.606041	1.487900	0.426521	H	3.152218	-2.182264	-2.567959
C	-5.949171	4.810755	-0.037848	C	1.561515	-5.149918	-1.981200
H	-3.984487	5.636713	-0.399474	H	0.336907	-5.568011	-0.248714
H	-7.768085	3.698206	0.317773	H	2.811004	-4.434431	-3.594601
H	-6.454057	5.779162	-0.085968	H	1.410098	-6.136356	-2.427813
C	-4.237624	-2.422239	-0.136668	C	2.777909	2.076181	-0.734267
C	-3.458635	-3.511914	-0.451013	C	2.403784	3.285522	-0.195563
S	-5.907702	-2.866142	-0.020038	S	3.611627	2.296644	-2.235484
C	-4.215894	-4.710205	-0.590649	C	2.801375	4.393870	-0.997616
H	-2.379965	-3.452351	-0.608162	H	1.830688	3.377937	0.729126
C	-5.553628	-4.506935	-0.383399	C	3.465055	4.004907	-2.129946
H	-3.782375	-5.680199	-0.840893	H	2.594656	5.435999	-0.746758
H	-6.356180	-5.243708	-0.430154	H	3.873480	4.640523	-2.916337
C	-1.376866	-1.328283	0.860355	C	2.485789	1.144350	2.338786
H	-1.562777	-2.282865	1.365210	H	3.139074	2.022226	2.259764
N	-0.139310	-1.035176	0.701549	N	1.989338	0.969182	3.509562
N	0.203063	0.093522	0.063225	N	1.127737	-0.086417	3.703929

H	-0.464374	0.596592	-0.522818	H	1.010174	-0.288204	4.691845
C	1.531735	0.345351	-0.191179	C	-0.035788	-0.282283	2.971213
S	2.783815	-0.631288	0.517193	S	-0.726581	0.940887	1.928856
C	3.296033	1.347187	-0.997714	C	-1.853857	-1.301506	2.281259
C	3.961669	0.387710	-0.286067	C	-2.035644	-0.149422	1.566464
H	3.788091	2.101587	-1.616012	H	-2.521055	-2.164801	2.225088
N	1.927321	1.325273	-0.940401	N	-0.726668	-1.377635	3.054067
C	5.408476	0.170613	-0.149927	C	-3.092990	0.189620	0.602740
C	5.951415	-1.112228	0.045554	C	-2.812394	0.909405	-0.572842
C	6.300030	1.246550	-0.222983	C	-4.411943	-0.218924	0.821720
C	7.318997	-1.305958	0.158424	C	-3.810745	1.201242	-1.487795
H	5.286704	-1.980363	0.097432	H	-1.787000	1.231107	-0.780470
C	7.679663	1.064267	-0.125116	C	-5.424721	0.059991	-0.097069
H	5.910483	2.261390	-0.343436	H	-4.662860	-0.756482	1.740727
C	8.201050	-0.219345	0.069725	C	-5.128926	0.777072	-1.261427
H	8.331833	1.937367	-0.186853	H	-6.440618	-0.276856	0.117045
H	7.738048	-2.303787	0.308575	H	-3.592931	1.754915	-2.404111
O	9.514698	-0.504635	0.186844	O	-6.031759	1.105257	-2.207783
C	10.450088	0.532614	0.091653	C	-7.367889	0.721635	-2.039618
H	10.312161	1.284423	0.892335	H	-7.810965	1.171741	-1.130742
H	11.441016	0.069280	0.205018	H	-7.910667	1.090601	-2.921936
H	10.399869	1.038984	-0.891332	H	-7.474754	-0.378730	-1.986392
		**4**				**4_{cis}**	
E(scf) = -2056.06229482 a.u.				E(scf) = -2056.05419211 a.u.			
C	-3.437881	-1.065927	-0.099593	C	2.359893	0.355373	-0.121841
C	-2.159057	-0.548409	-0.480316	C	2.063919	0.051205	1.242805
C	-2.365587	0.821584	-0.534848	C	1.574616	-1.243993	1.191567
N	-3.665438	1.045970	-0.222586	N	1.599515	-1.627251	-0.103047
H	-1.688580	1.630237	-0.800056	H	1.239965	-1.907639	1.984337
N	-4.317090	-0.089042	0.050126	N	2.072938	-0.669579	-0.910204
C	-4.338370	2.294789	-0.132612	C	1.128835	-2.855985	-0.635880
C	-3.808589	3.431539	-0.747400	C	0.118563	-3.555964	0.027491
C	-5.537176	2.371103	0.580168	C	1.673997	-3.343722	-1.824472
C	-4.473358	4.650501	-0.629345	C	-0.329679	-4.766928	-0.496219
H	-2.888078	3.376481	-1.332654	H	-0.335450	-3.148007	0.934685
C	-6.196436	3.593183	0.681274	C	1.208360	-4.549528	-2.342419
H	-5.938513	1.468977	1.043326	H	2.450513	-2.767487	-2.330445
C	-5.667693	4.737768	0.084095	C	0.211621	-5.267524	-1.679813
H	-4.053056	5.537665	-1.110674	H	-1.122480	-5.313990	0.021354
H	-7.135236	3.649646	1.239141	H	1.634145	-4.934045	-3.273401
H	-6.186991	5.695703	0.171692	H	-0.149057	-6.214128	-2.091318
C	-3.821270	-2.457545	0.117615	C	2.874316	1.605960	-0.673140
C	-3.028094	-3.530695	0.453113	C	2.704123	2.886721	-0.200305
S	-5.481639	-2.931034	-0.018761	S	3.810414	1.600714	-2.128797
C	-3.766714	-4.740480	0.593296	C	3.340057	3.865592	-1.017216

H	-1.953029	-3.451450	0.625386	H	2.112896	3.124092	0.686537
C	-5.104664	-4.561783	0.365363	C	3.976970	3.309923	-2.094119
H	-3.320518	-5.700669	0.858568	H	3.316548	4.938302	-0.816001
H	-5.895136	-5.311783	0.407070	H	4.535697	3.822763	-2.877758
C	-0.968961	-1.319987	-0.846925	C	2.284351	0.896786	2.412861
H	-1.132050	-2.285749	-1.338396	H	3.101080	1.626022	2.345120
N	0.261378	-1.004723	-0.678112	N	1.682979	0.894441	3.547446
N	0.580107	0.140341	-0.054653	N	0.619725	0.041700	3.728411
H	-0.098465	0.643972	0.517850	H	0.393612	-0.068511	4.711702
C	1.903258	0.413634	0.199625	C	-0.488673	-0.017376	2.896992
S	3.165485	-0.579443	-0.467211	S	-0.888075	1.238949	1.745474
C	3.655719	1.460340	0.973138	C	-2.365857	-0.795503	2.065180
C	4.331047	0.482895	0.295480	C	-2.300154	0.329459	1.289578
H	4.139292	2.270568	1.523980	H	-3.145045	-1.555799	1.972775
N	2.288349	1.419856	0.919726	N	-1.343211	-0.993501	2.951242
C	5.780552	0.275327	0.170926	C	-3.190560	0.755456	0.199057
C	6.340111	-0.327825	-0.966413	C	-2.683979	1.359171	-0.962351
C	6.648558	0.692515	1.193244	C	-4.573632	0.539630	0.292770
C	7.715313	-0.506863	-1.087055	C	-3.529832	1.736304	-2.001132
H	5.689451	-0.654605	-1.783209	H	-1.606869	1.521999	-1.065858
C	8.026100	0.532507	1.079202	C	-5.428466	0.902146	-0.744310
H	6.237506	1.136253	2.103870	H	-4.989976	0.094079	1.200311
C	8.544311	-0.068227	-0.062125	C	-4.894113	1.499370	-1.879829
H	8.704685	0.855071	1.872281	H	-6.506658	0.739719	-0.677200
H	8.152954	-0.974939	-1.971765	H	-3.141603	2.202544	-2.909478
F	9.862928	-0.231580	-0.172424	F	-5.708489	1.856150	-2.872296

**5**				**5_{cis}**			
E(scf) = -2416.41832623 a.u.				E(scf) = -2416.41007845 a.u.			
C	-3.792866	-1.073043	-0.103379	C	-2.495822	-0.732796	-0.212962
C	-2.515428	-0.550403	-0.481469	C	-2.339368	-0.288368	1.136276
C	-2.727645	0.818680	-0.538258	C	-2.093273	1.069956	1.008512
N	-4.029135	1.037714	-0.229651	N	-2.120907	1.355582	-0.310987
H	-2.053447	1.630035	-0.802487	H	-1.933255	1.835320	1.763293
N	-4.676609	-0.099721	0.042963	N	-2.362348	0.274087	-1.062313
C	-4.707785	2.283786	-0.143465	C	-1.902147	2.619839	-0.919530
C	-4.184284	3.420361	-0.763838	C	-1.113979	3.577428	-0.277669
C	-5.905632	2.357233	0.571144	C	-2.478231	2.886252	-2.162528
C	-4.854395	4.636746	-0.649368	C	-0.925400	4.820577	-0.879011
H	-3.264734	3.366824	-1.350789	H	-0.628990	3.350428	0.675424
C	-6.570345	3.576656	0.668553	C	-2.271856	4.128074	-2.757026
H	-6.302036	1.455034	1.038422	H	-3.075227	2.113136	-2.648946
C	-6.047827	4.721253	0.065923	C	-1.502391	5.100781	-2.117178
H	-4.439119	5.523942	-1.134968	H	-0.307713	5.570177	-0.376924
H	-7.508438	3.631065	1.227803	H	-2.723090	4.337798	-3.730730
H	-6.571348	5.677146	0.150631	H	-1.346559	6.075356	-2.587560

C	-4.170545	-2.466032	0.114660	C	-2.735921	-2.089873	-0.695042
C	-3.373046	-3.535545	0.451565	C	-2.372584	-3.284969	-0.118334
S	-5.828792	-2.946502	-0.022416	S	-3.546068	-2.349032	-2.202831
C	-4.106720	-4.748261	0.592364	C	-2.758907	-4.413652	-0.897018
H	-2.298445	-3.451693	0.624601	H	-1.815396	-3.353983	0.818160
C	-5.445257	-4.575286	0.363383	C	-3.403629	-4.054081	-2.049830
H	-3.656755	-5.706358	0.858813	H	-2.558003	-5.448944	-0.615197
H	-6.232679	-5.328469	0.405293	H	-3.800626	-4.709958	-2.825368
C	-1.321168	-1.316982	-0.844981	C	-2.493407	-1.078744	2.353618
H	-1.478756	-2.282264	-1.339266	H	-3.150061	-1.955185	2.286119
N	-0.092793	-0.997750	-0.669924	N	-2.010350	-0.881076	3.526378
N	0.217750	0.146803	-0.040497	N	-1.143379	0.173280	3.706735
H	-0.466180	0.645630	0.529883	H	-1.059098	0.423953	4.686842
C	1.537727	0.426784	0.218170	C	0.036149	0.346951	3.003356
S	2.807315	-0.554371	-0.451405	S	0.707977	-0.868291	1.940886
C	3.280790	1.479293	1.003972	C	1.902020	1.323784	2.386179
C	3.964345	0.509561	0.322351	C	2.062560	0.185856	1.643191
H	3.757209	2.289066	1.561591	H	2.597395	2.166317	2.370467
N	1.914793	1.431671	0.945445	N	0.762335	1.416535	3.134633
C	5.414364	0.313139	0.199664	C	3.123335	-0.151980	0.684019
C	5.977393	-0.317934	-0.920221	C	2.851047	-0.899123	-0.472072
C	6.282410	0.767327	1.205732	C	4.437211	0.292275	0.895559
C	7.353581	-0.487359	-1.039496	C	3.853723	-1.189116	-1.392134
H	5.329241	-0.675102	-1.726285	H	1.832249	-1.246667	-0.669059
C	7.660356	0.615293	1.092065	C	5.445355	0.017850	-0.023578
H	5.872624	1.233633	2.105560	H	4.679991	0.849788	1.804342
C	8.192188	-0.014088	-0.032551	C	5.148086	-0.724147	-1.165723
H	8.325571	0.971942	1.881512	H	6.465464	0.367669	0.149253
H	7.778320	-0.978580	-1.917602	H	3.630646	-1.766074	-2.292167
Cl	9.916075	-0.215646	-0.175590	Cl	6.405226	-1.075145	-2.317906
		**6**				$\mathbf{6_{cis}}$	
E(scf) = -4530.42175374 a.u.				E(scf) = -4530.41369373 a.u.			
C	-4.504111	-1.081474	-0.107426	C	-2.852730	-0.952321	-0.332673
C	-3.226618	-0.552819	-0.476704	C	-2.920255	-0.361820	0.967127
C	-3.444896	0.815257	-0.535322	C	-2.802502	0.996390	0.717235
N	-4.749503	1.028083	-0.235950	N	-2.687022	1.145274	-0.619523
H	-2.772684	1.629841	-0.794701	H	-2.819869	1.844987	1.395836
N	-5.393476	-0.112318	0.032554	N	-2.715259	-0.025663	-1.268735
C	-5.434880	2.270891	-0.156031	C	-2.503247	2.362181	-1.327542
C	-4.915360	3.407875	-0.778957	C	-1.881289	3.442777	-0.698326
C	-6.635209	2.340577	0.554721	C	-2.943075	2.461066	-2.648447
C	-5.592042	4.621193	-0.670772	C	-1.724611	4.639807	-1.394042
H	-3.993950	3.356665	-1.363216	H	-1.498279	3.345906	0.321184
C	-7.306507	3.556882	0.645807	C	-2.769167	3.659593	-3.335565
H	-7.028404	1.437954	1.023924	H	-3.410053	1.595185	-3.120766

C	-6.788022	4.702012	0.040690
H	-5.179987	5.508798	-1.158362
H	-8.246527	3.608481	1.202071
H	-7.316725	5.655485	0.120362
C	-4.876679	-2.476205	0.108077
C	-4.076259	-3.542337	0.448750
S	-6.531993	-2.963956	-0.038405
C	-4.805308	-4.758377	0.584884
H	-3.002965	-3.453860	0.627506
C	-6.143338	-4.591196	0.348696
H	-4.352598	-5.714625	0.853321
H	-6.927673	-5.347823	0.385992
C	-2.026492	-1.313440	-0.833310
H	-2.176464	-2.278669	-1.330110
N	-0.800831	-0.988636	-0.649919
N	-0.500282	0.156086	-0.015540
H	-1.190159	0.649038	0.552799
C	0.816574	0.443705	0.249248
S	2.094891	-0.523689	-0.423362
C	2.549618	1.503654	1.046999
C	3.242126	0.543390	0.360720
H	3.018564	2.313000	1.611520
N	1.184420	1.446694	0.984145
C	4.693498	0.358028	0.239419
C	5.261417	-0.267629	-0.880879
C	5.557839	0.816529	1.246576
C	6.638994	-0.427978	-0.999290
H	4.616617	-0.627835	-1.688292
C	6.937177	0.673179	1.133748
H	5.144900	1.279206	2.146859
C	7.474706	0.048594	0.008797
H	7.593995	1.035525	1.928039
H	7.061156	-0.916020	-1.880646
Br	9.348197	-0.159545	-0.146471

C	-2.167574	4.753357	-2.711432
H	-1.237688	5.486401	-0.902374
H	-3.113565	3.740173	-4.370338
H	-2.037424	5.693337	-3.254361
C	-2.884411	-2.370849	-0.677469
C	-2.451088	-3.443496	0.067505
S	-3.494705	-2.887600	-2.212259
C	-2.620821	-4.687769	-0.605120
H	-1.991143	-3.340774	1.052551
C	-3.175444	-4.535264	-1.847472
H	-2.334901	-5.653558	-0.184296
H	-3.411222	-5.313022	-2.574505
C	-3.143628	-1.037863	2.241379
H	-3.677140	-1.995657	2.200054
N	-2.842971	-0.659645	3.430937
N	-2.134715	0.514334	3.577157
H	-2.200870	0.873623	4.524408
C	-0.900025	0.744672	2.992826
S	0.049024	-0.518314	2.242597
C	0.896347	1.865258	2.414170
C	1.289389	0.656882	1.906303
H	1.477399	2.785900	2.322732
N	-0.334452	1.914111	3.007144
C	2.501060	0.317618	1.147203
C	2.479374	-0.655854	0.137224
C	3.710752	0.977881	1.408987
C	3.625291	-0.962388	-0.590333
H	1.545721	-1.175433	-0.099005
C	4.860389	0.686794	0.680298
H	3.760188	1.721509	2.209007
C	4.813408	-0.286460	-0.316967
H	5.795906	1.207403	0.897521
H	3.590184	-1.721153	-1.375171
Br	6.373649	-0.698292	-1.302456

2_{iso}

E(scf) = -2161.32995310 a.u.

C	-3.809182	1.127668	0.094900
C	-2.547367	0.555124	0.451463
C	-2.829269	-0.795461	0.590405
N	-4.151940	-0.957222	0.343776
H	-2.189644	-1.629653	0.870070
N	-4.748549	0.198328	0.034274
C	-4.899503	-2.166344	0.349755
C	-4.439510	-3.280265	1.055258
C	-6.100437	-2.224567	-0.360660

C	-5.177379	-4.462265	1.029434	
H	-3.518887	-3.232231	1.641284	
C	-6.833305	-3.408206	-0.368907	
H	-6.445363	-1.337811	-0.894031	
C	-6.374672	-4.532118	0.318937	
H	-4.813469	-5.332673	1.582044	
H	-7.773902	-3.451512	-0.924865	
H	-6.951841	-5.460470	0.305429	
C	-4.118476	2.526409	-0.184598	
C	-3.275204	3.529126	-0.604989	
S	-5.741136	3.107790	-0.018017	
C	-3.944944	4.772902	-0.786967	
H	-2.213596	3.375332	-0.807733	
C	-5.281894	4.689233	-0.504416	
H	-3.453234	5.688289	-1.121178	
H	-6.028050	5.482394	-0.561356	
C	-1.298384	1.271591	0.722379	
H	-1.380255	2.259333	1.190204	
N	-0.099699	0.886556	0.486864	
N	0.116328	-0.292359	-0.120795	
H	-0.627411	-0.781301	-0.620658	
C	1.400269	-0.638275	-0.456966	
S	2.750806	0.312655	0.084208	
C	3.037905	-1.786105	-1.329355	
C	3.805516	-0.821102	-0.734773	
H	3.439880	-2.632517	-1.891341	
N	1.684764	-1.682706	-1.173143	
C	5.266209	-0.679668	-0.729799	
C	6.040427	-1.219696	-1.771833	
C	5.928061	-0.013088	0.310152	
C	7.428771	-1.114502	-1.768350	
H	5.542994	-1.714981	-2.609733	
C	7.313100	0.090105	0.284919	
H	5.382475	0.420740	1.150133	
C	8.086670	-0.452225	-0.734511	
H	8.006347	-1.543332	-2.591303	
H	9.171369	-0.345381	-0.709414	
O	7.292123	1.265973	2.264912	
N	7.988077	0.797543	1.391698	
O	9.196701	0.865033	1.355814	

3. Molecular Docking

Molecular docking studies were conducted to understand the binding interactions of the synthesized compounds with potential biological targets. The docking results revealed that the compounds fit well into the active sites of the target proteins, forming stable interactions that support their observed antimicrobial. The binding affinities and interaction details provided insights into the mechanisms of action of the compounds, suggesting that they could serve as leads for further drug development.

Molecular docking studies of compounds **7a-g** and penicillin as the reference drug were performed with penicillin-binding proteins from gram-positive (*S. aureus y B. subtillis*) and gram-negative (*E. coli*) bacteria were performed. Fluconazole as a reference drug on the fungal protein sterol 14α-demethylase of *C. albicans*; all pathogenic microorganisms of medical interest. To understand the affinity with which ligands bind at the active site of the proteins, the affinity energy was determined as show in Figure 3. A more negative value indicates a higher ligand-receptor binding affinity; however, to have a pharmacological affect in addition to affinity, interaction with specific amino acids is also required to inhibit or activate pharmacological targets, as explained in Figure 4. Where the binding modes of compounds **7d** and **7g** in the active sites of bacterial proteins are described, these two molecules being the ones that presented the best inhibitory activity according to the results shown in Table 1 and Figure 1 (Manuscript). Likewise, the interactions of **7g** on sterol 14α-demethylase, a compound with the best fungal inhibition activity, are described.

Methicillin-resistant *S. aureus* (MRSA) infection is a leading cause of mortality and morbidity among bacterial diseases. Penicillin-binding protein (PBP) play a primary role in building the bacterial cell wall. In this sense, MRSA expresses PBP2a (Figure 4A1) resistant to the action of methicillin and other b-lactams; protein on which molecular docking were carried out. Figure 3 (Manuscript) show the affinity energies (ΔG=*kcal/mol*) of the binding of compounds **7a-g** and penicillin at the active site of PBP2a, observing that penicillin has an affinity energy of -8.18 *kcal*/mol and the compounds under study have similar energies (-8.14, -7.89, -7.92, -8.21, -8.08, -8.10 and **-**8.15 respectively **7a-g**), it should be noted that compounds **7d** and g are the ones with the

best affinity energies (Table S2), which is consistent with the inhibitory activity on this microorganism reported in Table 2 (Manuscript).

Table S2. Affinity energy (ΔG, *kcal/mol*) of the molecules under study at the active site of bacterial penicillin-binding protein and fungal Lanosterol 14α-demethylase

Molecule	*S. aureus* 1MWT	*B. subtillis* 1W5D	*E. coli* 2EX8	*C. albicans* 5TZ1
**7a**	-8.14	-8.76	-8.07	-10.38
**7b**	-7.89	-8.22	-7.56	-10.35
**7c**	-7.92	-7.95	-7.25	-10.33
**7d**	-8.21	-8.09	-7.83	-10.40
**7e**	-8.08	-8.19	-7.91	-9.94
**7f**	-8.10	-8.16	-7.86	-10.25
**7g**	-8.15	-8.30	-8.05	-10.49
Penicillin	-8.18	-8.90	-8.24	-----
Fluconazole	-----	-----	-----	-8.33

According to Lim and Strynadka,[9] the amino acids that form the active site of PBP2a are Ser403, Lys406, Tyr446, Ser462, Asn464, Ser598, Gly599, Thr600 y Met641; Ser403 being the catalytic base with which inhibitors interact. Figure 4A2 show that penicillin interacts at the active site with the amino acids Ser403, Lys406, Ser462, Ser598 with hydrogen bonding interaction, while Tyr446 and Met641 with a π-sulfur interaction. In the case of compounds **7d** (Figure 4A3) it interacts through a hydrogen bond with the Ser462 residue, with interaction of a π-donor hydrogen bond with the amino acids Tyr446 and Asn464, while Thr600 with a C-H bond and π-π stacking, also with Met641 whit π-π interaction. The molecule **7g** (Figure 4A4) interacts with H-bond with the amino acids Ser403 and Tyr446, Asn464 interacts with the ligand with a π-donor hydrogen bond and Thr600 with a C-H bond and the Met641 residue with π-π stacks.

To understand the binding mode of penicillin and **7a-g** compounds in the active site of the penicillin-binding protein (PBP) of *E. coli*, PBP4 (Figure 4B1) was used, which has both endopeptidase and carboxypeptidase activity, which is one of the 12 PBPs that participate in the synthesis and maintenance of the cell wall of the pathogen. We evaluated the affinity of penicillin on the active site of PBP4 by finding a binding energy of -8.24 *kcal*/mol while the values of the binding affinities of the **7a-g** compounds are similar (-8.07, -7.56, -7.25, -7.83, -7.91, -7.86, -8.05 *kcal/mol*, respectively), as shown in Figure 3.

According to Kishida, H., et al.,[10] the active site is made up of the residues Ser62, Asn154, Asp155, Met156, Thr157, Gln158, Cys159, Phe160, Ser161, Lys305, Ser306, Asp307, Asn308, Gly419, Ser420, Leu421. In this sense, Figure 4B2 shows the mode of binding of penicillin at the active site of PBP4, it interacts with hydrogen bonds, the main residue Ser62, and with the amino acids Lys65, Ser306, Asn308, Ser398 and Lys417. It also interacts with Gly419 and Ser420 with carbon hydrogen bonds. Similarly, **7d** (Figure 4B3) interacts with Ser62 by hydrogen bonds, with π-π stacking with Phe160, π-cation interaction with Arg361, with Leu359 and Leu421 through π-alkyl interactions, with carbon hydrogen bonds with the Gly419 and Thr418 residue, while Gln422 interacts with a π-donor hydrogen bond and Ser398 presents a halogen bridge. Figure 4B4 shows the interactions of **7g**, it forms a H-bond with Ser62, π-π stacking with Phe160, while with Leu421 it is through π-alkyl interactions, π-cation interaction with Arg361, with a π-donor H-bond, it bonds with Thr418 and Gly419 with C-H bonds.

In Figure 3, we can see that penicillin has a discrete but higher affinity energy (-8.90 *kcal*/mol) compared to the **7a-g** compounds under study (-8.76, -8.22, -7.95, -8.09, -8.19, -8.16 y -8.30 *kcal*/mol, respectively), when interacting at the active site of the penicillin-binding protein of *B. subtillis* PBP4a (Figure 4C1), which, it may be related to their increased biological activity.

The active site PBP4a is formed by the residues Ser52, Lys55, Asp145, Tyr150, Ser299, Asn301, His352, Thr394, Arg398, Lys411, Thr412 and Ser414.[11] It should be noted that the central amino acid is Ser52 and that it is important in the inhibition of this protein. Figure 4C2 show the interactions of penicillin at the active site of PBP4a. The residues Ser52, Lys55, Asn301, Lys411, Thr412, Lys449 by hydrogen bonds,

while by carbon hydrogen bond interacts with Thr394, Ser414 and Ser299, with the latter amino acid also presents a π-lone pair interaction, with Tyr149 it forms a π-donor hydrogen bond.

The binding mode and interactions of ligand **7d** at the active site of PBP4a are shown in Figure 4C3. It interacts with Ser52 forming a H-bond and a carbon hydrogen bond, with Thr149 it forms a π-donor H-bond and a π-π stacking with Tyr150, with Ser414 a sulphur-x interaction is formed that occurs between the divalent sulphur and O, N or S atoms, while it joins with a halogen bridge with Thr394. The **7g** compound's bond in similar manner as seen in Figure 4C4. It interacts with Ser52 by forming H-bonds and a C-H bond, with Tyr149 through a π-donor H-bond, and with a π-π stacking with Tyr150, in the same way that **7d** forms a sulphur-x interaction with Ser414.

To understand the antifungal biological activity of synthesized compounds **7a-g** and fluconazole as a reference, the binding mode at the active site of the protein sterol 14α-demethylase or CYP51 of *C. albicans* was analysed (Fig. 4D1). It is interesting that **7a-g** compounds have better affinity energy (ΔG) (-10.38, -10.35, -10.33, -10.40, -9.94, -10.25 and -10.49 *kcal/mol*, respectively), compared to fluconazole (-8.33 *kcal/mol*) as shown in Figure 3. This binding affinity is influenced by the number of ligand-receptor interactions and as show in Figure 4, **7g** has a higher number of interactions than fluconazole, which is reflected in its ΔG. However, another factor that is involved in biological activity is interactions with specific amino acids or cofactor.

In this sense, it should be noted that azoles that inhibit CYP51 activity interact with iron from the heme prosthetic group.[12] Likewise, the active site of CYP51 is formed by the amino acids of Glu116-Pro135, Leu220-Gly227, Tyr243-Ile253, Ile297-Ser314, Met374-Arg381 and Asp504-Glu514.[13] Figure 4D2 show that fluconazole binds with π-alkyl interactions with Ile131, Ile304, Leu376 and with the iron of the heme group (distance 3.77 Å), in the same way it bind to the hem group by π-donor H-bond, while with Tyr132 it binds with a H-bond and by π-π stacking.

Figure 4D3 shows the binding mode of the **7g** ligand, which is the compound with the best antifungal activity in the set under study, as reported in Figure 1. **7g** interacts with Tyr118 by π-π stacking and with Try132 with a H-bond, while with Ile131, Leu376,

His377, Phe380, Met508 and Val509 with π-alkyl interactions, with Gly307 through a C-H bond. The **7g** thiophene ring is bound with the heme group by π-alkyl interactions, while the thiazole ring forms a H-bond, it also interacts with the iron of the heme group with the pyrazole ring by π-alkyl interaction (distance 5.32 Å).

Although both compounds interact with both the heme group and iron, the interaction presented by fluconazole occurs at a more favourable distance than that of the **7g** molecule; possibly resulting in its increased antifungal activity. However, **7g** molecules; possibly resulting in its increased antifungal activity. However, **7g**, as it presents a good biological activity, serves as a template for structural modifications and thus increases its antifungal potential. Also, thanks to its structure, it can reduce the toxicity of azoles used in clinical therapy.

**Table S3.** Molecular docking methods selected by re-docking for RMSD value less than 2 Å			
**Protein**	**Scoring Function**	**Search Algorithm**	**RMSD**
PBP2a-1MWT *S. aureus*	MoldDock Score	MoldDock SE	1.24
PBP4-2EX8 *E. coli*	MoldDock Score	MoldDock Optimaizer	1.63
PBP4a-1W5D *B. subtillis*	MoldDock Score	MoldDock Optimaizer	1.84
CYP51-5TZ1 *C. albicans*	MoldDock Score GRID	MoldDock SE	1.57

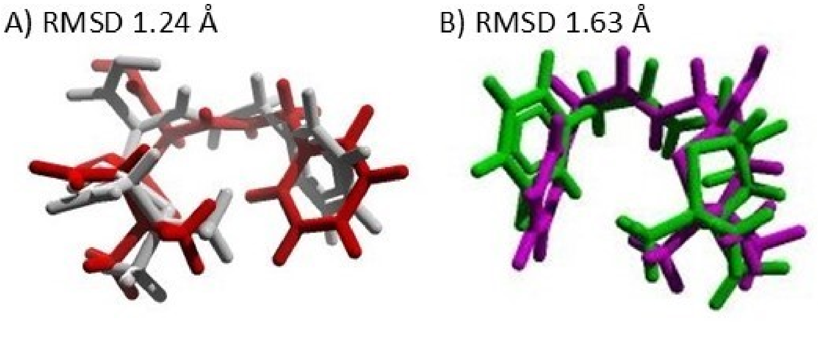

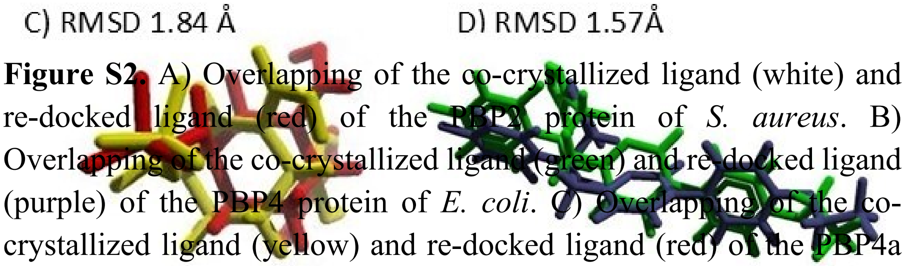

Figure S2. A) Overlapping of the co-crystallized ligand (white) and re-docked ligand (red) of the PBP2 protein of *S. aureus*. B) Overlapping of the co-crystallized ligand (green) and re-docked ligand (purple) of the PBP4 protein of *E. coli*. C) Overlapping of the co-crystallized ligand (yellow) and re-docked ligand (red) of the PBP4a protein of *B. subtillis*. D) Overlapping of the co-crystallized ligand (green) and re-docked ligand (blue) of the Sterol 14α-demethylase protein of *C. albicans*.

4. NMR Spectra

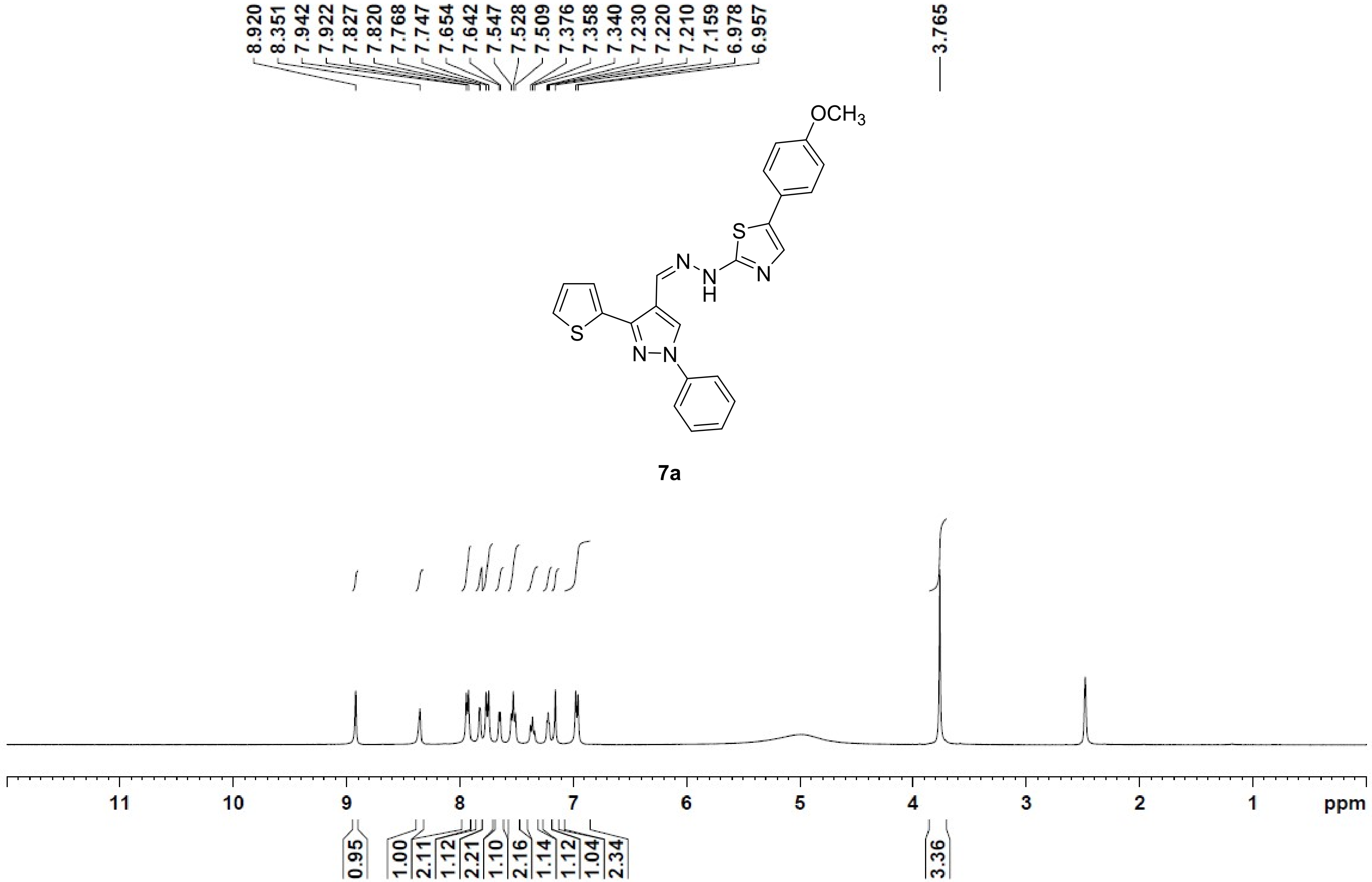
8.920 8.351 7.942 7.922 7.827 7.820 7.768 7.747 7.654 7.642 7.547 7.528 7.509 7.376 7.358 7.340 7.230 7.220 7.210 7.159 6.978 6.957
3.765
OCH3
S
N
N
H
N
S
N
N
7a
11 10 9 8 7 6 5 4 3 2 1 ppm
0.95 1.00 2.11 1.12 2.21 1.10 2.16 1.14 1.12 1.04 2.34
3.36

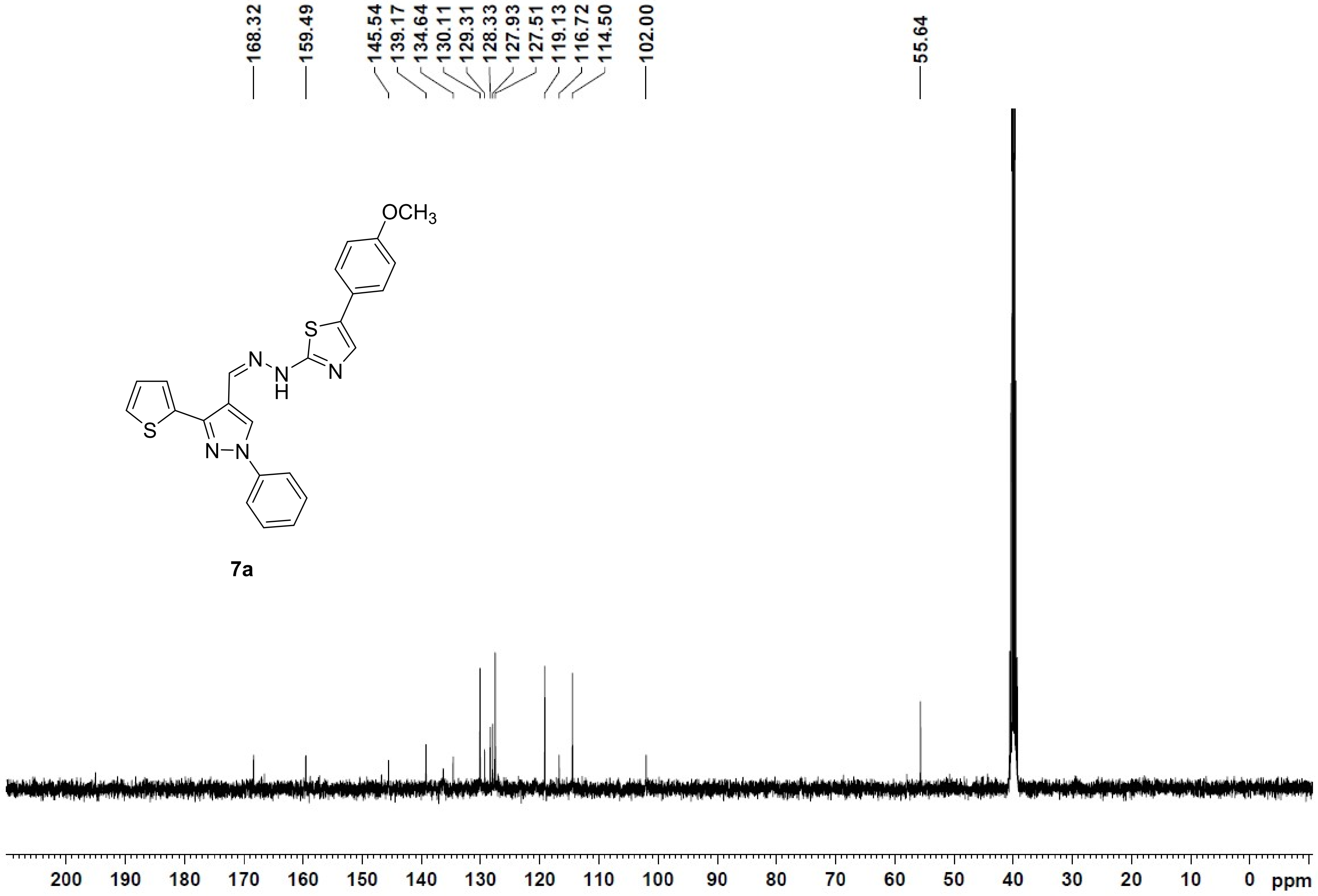

168.32
159.49
145.54
139.17
134.64
130.11
129.31
128.33
127.93
127.51
119.13
116.72
114.50
102.00
55.64
OCH3
S
N
N
H
S
N
N
N
200 190 180 170 160 150 140 130 120 110 100 90 80 70 60 50 40 30 20 10 0 ppm

7a

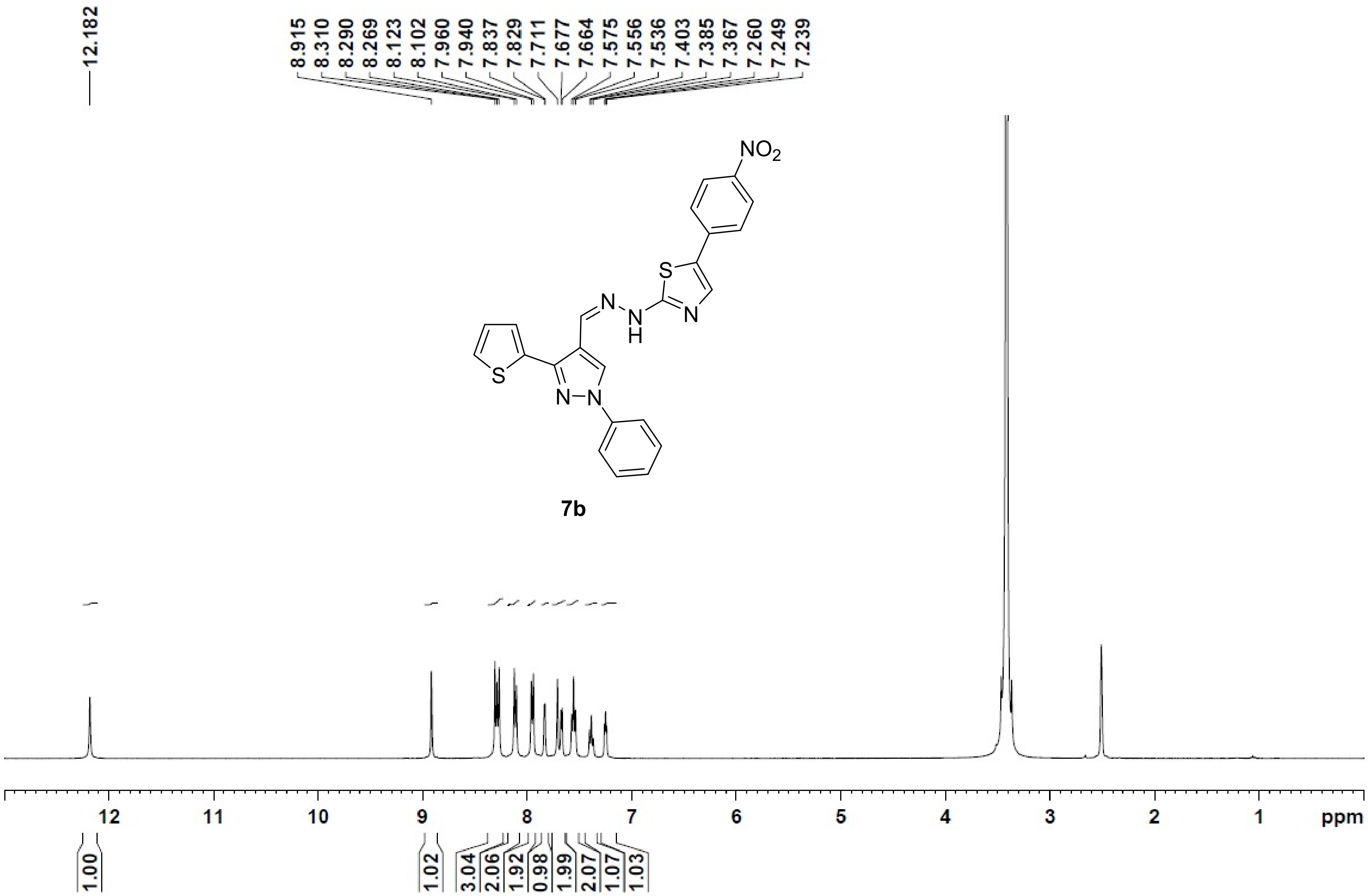
12.182
8.915
8.310
8.290
8.269
8.123
8.102
7.960
7.940
7.837
7.829
7.711
7.677
7.664
7.575
7.556
7.536
7.403
7.385
7.367
7.260
7.249
7.239
NO_2
S
N
N
H
S
N
N
7b
12
11
10
9
8
7
6
5
4
3
2
1
ppm
1.00
1.02
3.04
2.06
1.92
0.98
1.99
2.07
1.07
1.03

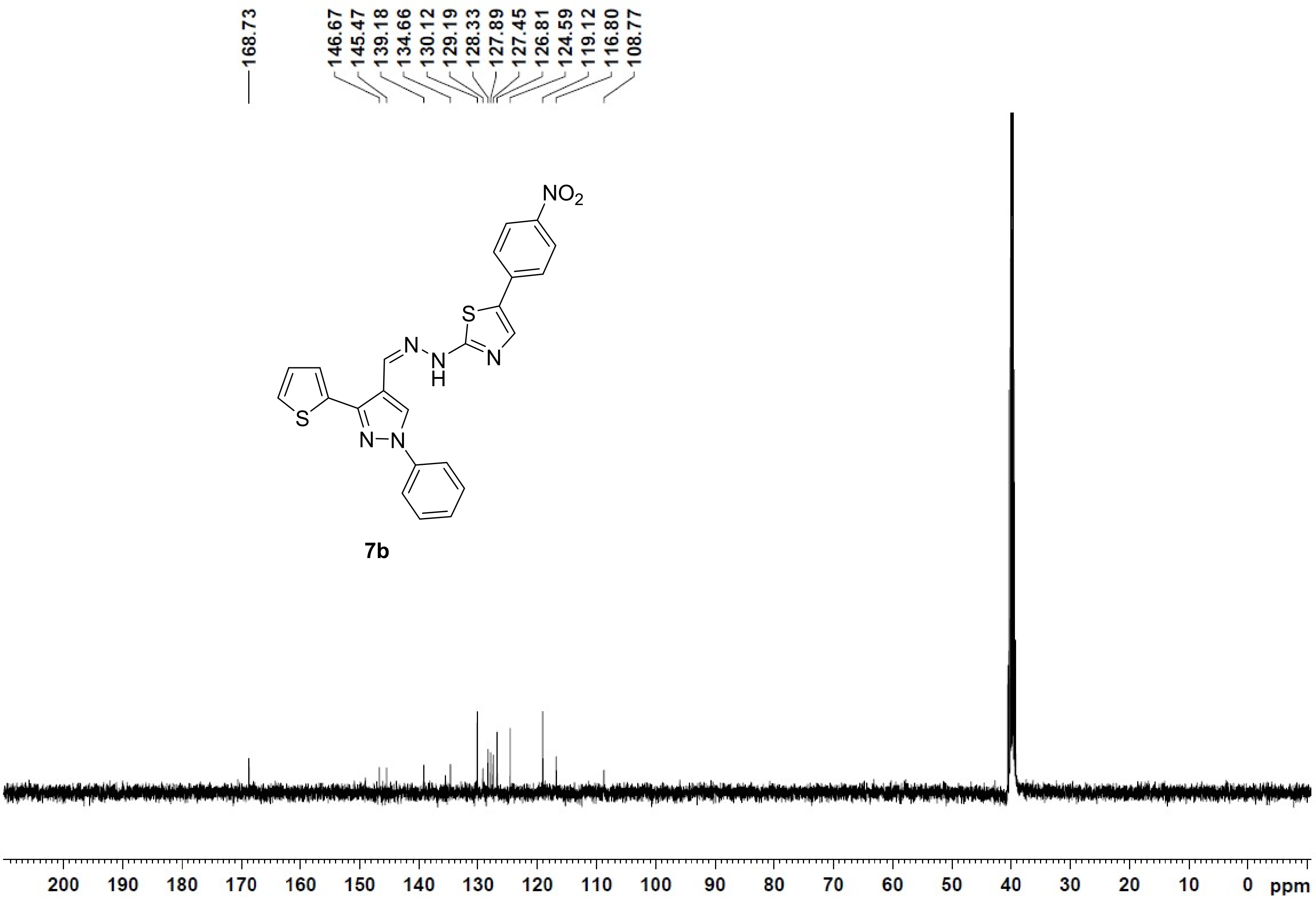
168.73
146.67
145.47
139.18
134.66
130.12
129.19
128.33
127.89
127.45
126.81
124.59
119.12
116.80
108.77
NO2
S
N
N
N
H
S
N
N
S
7b
200 190 180 170 160 150 140 130 120 110 100 90 80 70 60 50 40 30 20 10 0 ppm

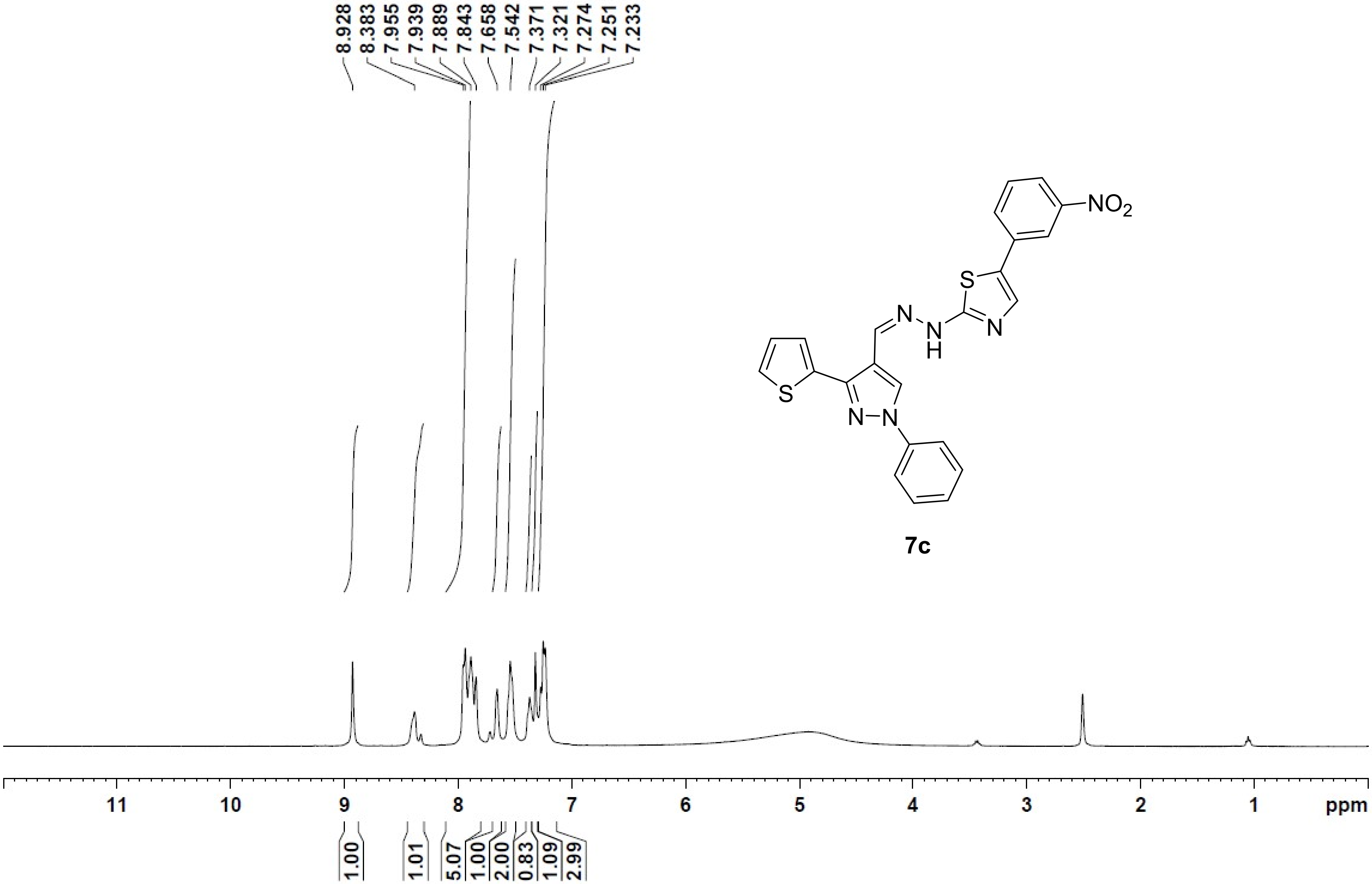
8.928
8.383
7.955
7.939
7.889
7.843
7.658
7.542
7.371
7.321
7.274
7.251
7.233
11
10
9
8
7
6
5
4
3
2
1
ppm
1.00
1.01
5.07
1.00
2.00
0.83
1.09
2.99
NO_2
S
N
N
H
N
S
N
N

7c

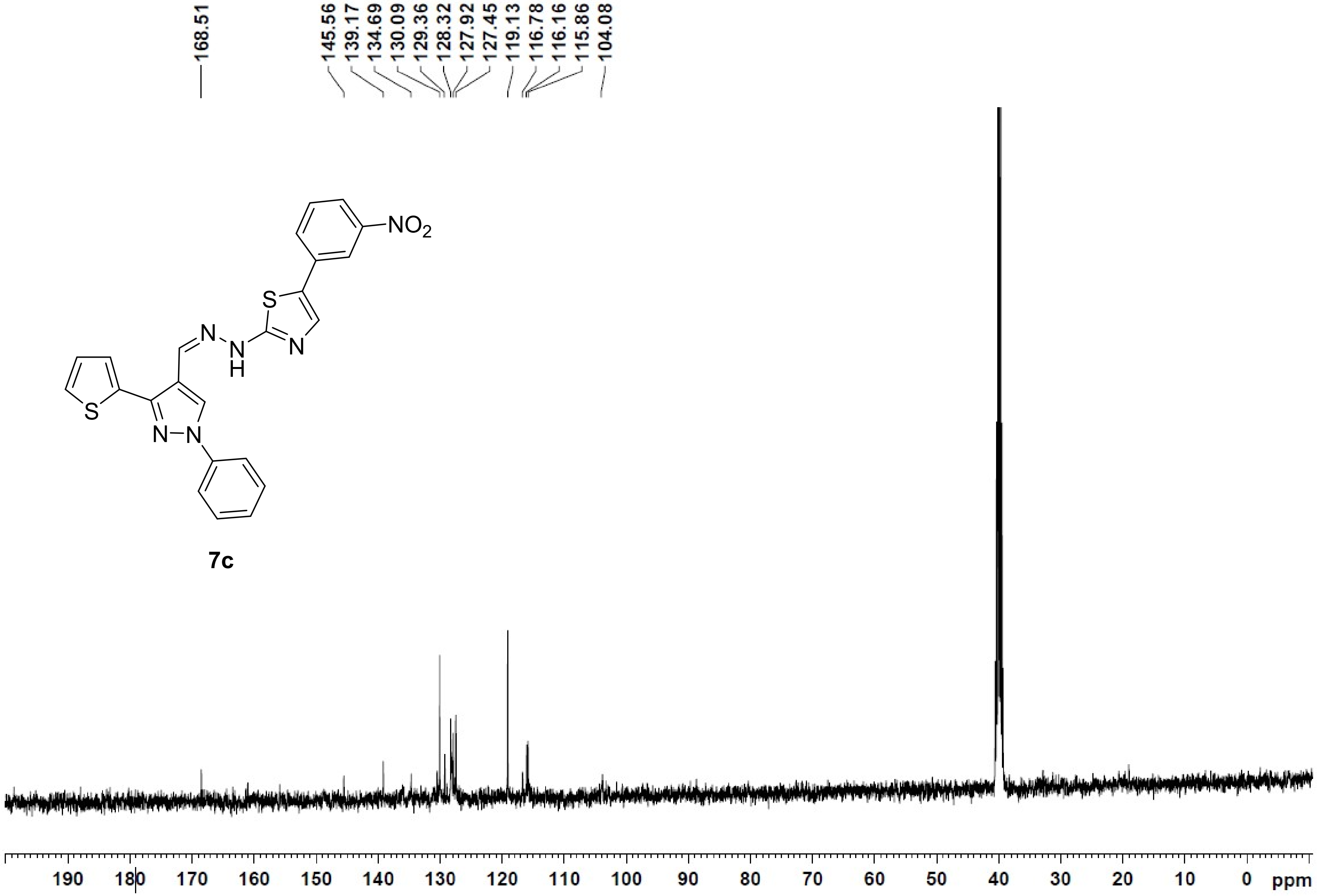
168.51
145.56
139.17
134.69
130.09
129.36
128.32
127.92
127.45
119.13
116.78
116.16
115.86
104.08
NO_2
S
N
N
H
S
N
N
S
190 180 170 160 150 140 130 120 110 100 90 80 70 60 50 40 30 20 10 0 ppm

7c

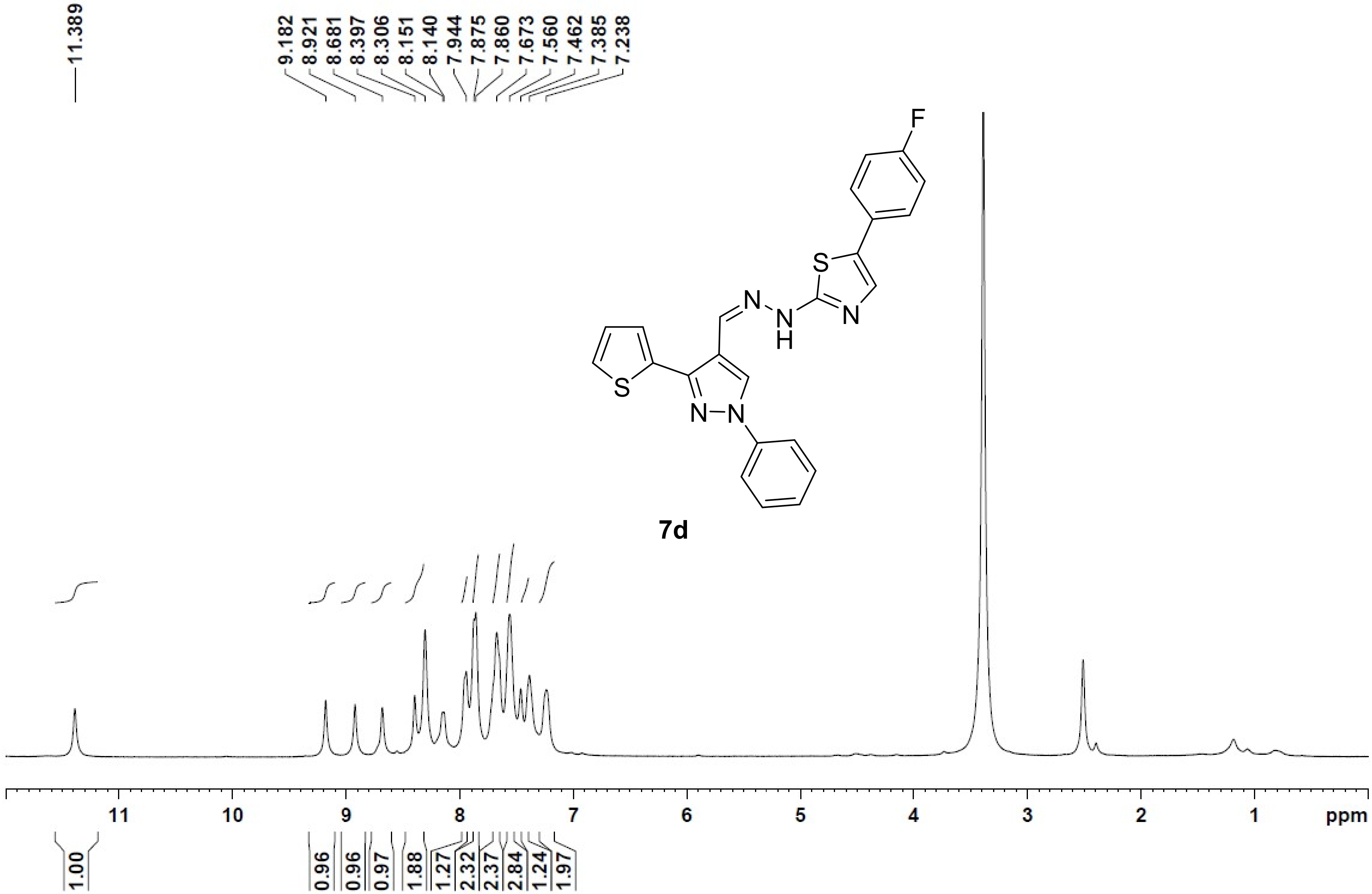
11.389
9.182
8.921
8.681
8.397
8.306
8.151
8.140
7.944
7.875
7.860
7.673
7.560
7.462
7.385
7.238
F
S
N
N
H
N
S
N
N
7d
11
10
9
8
7
6
5
4
3
2
1
ppm
1.00
0.96
0.96
0.97
1.88
1.27
2.32
2.37
2.84
1.24
1.97

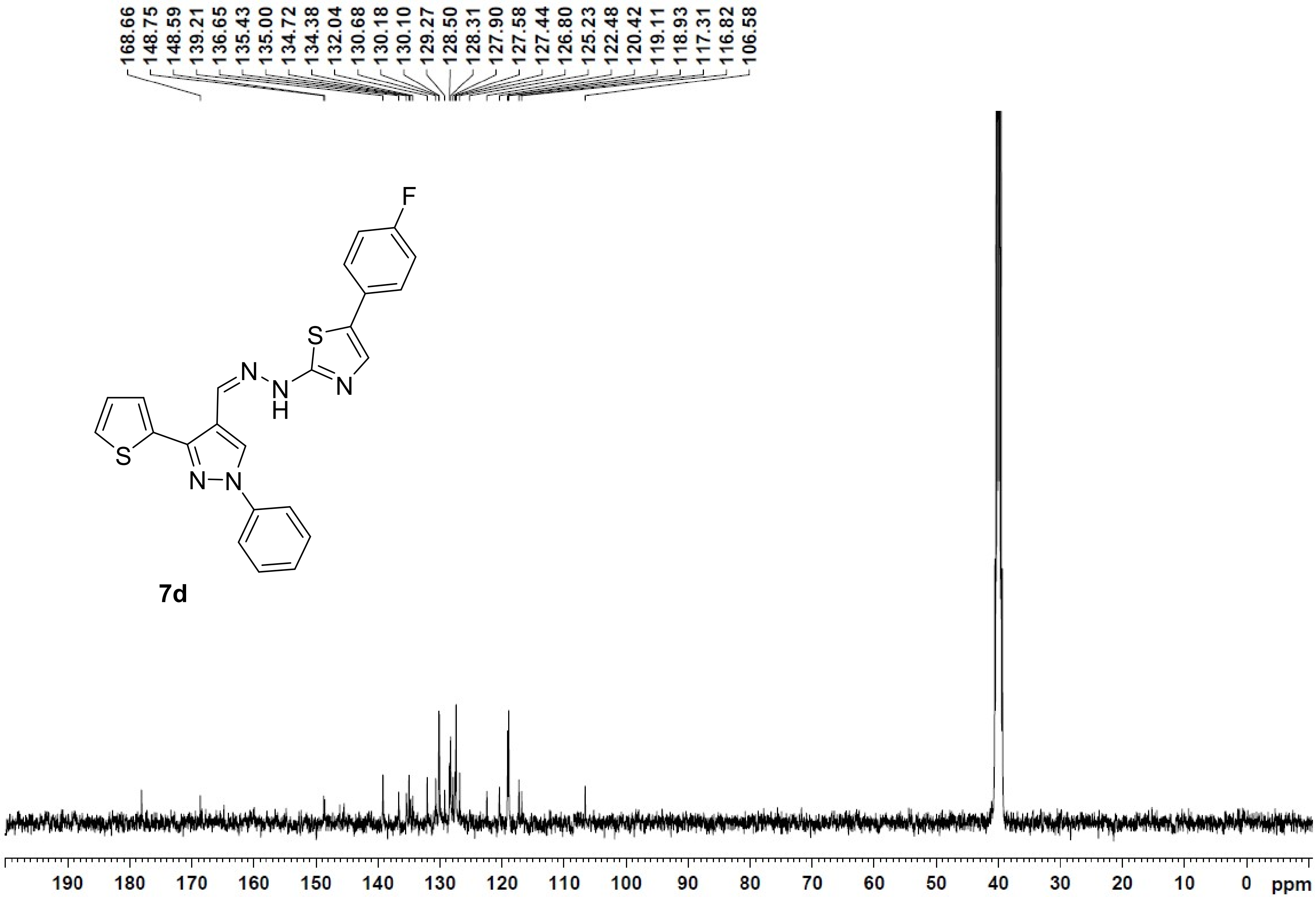
168.66
148.75
148.59
139.21
136.65
135.43
135.00
134.72
134.38
132.04
130.68
130.18
130.10
129.27
128.50
128.31
127.90
127.58
127.44
126.80
125.23
122.48
120.42
119.11
118.93
117.31
116.82
106.58
F
S
N
N
H
N
S
N
N
190 180 170 160 150 140 130 120 110 100 90 80 70 60 50 40 30 20 10 0 ppm

7d

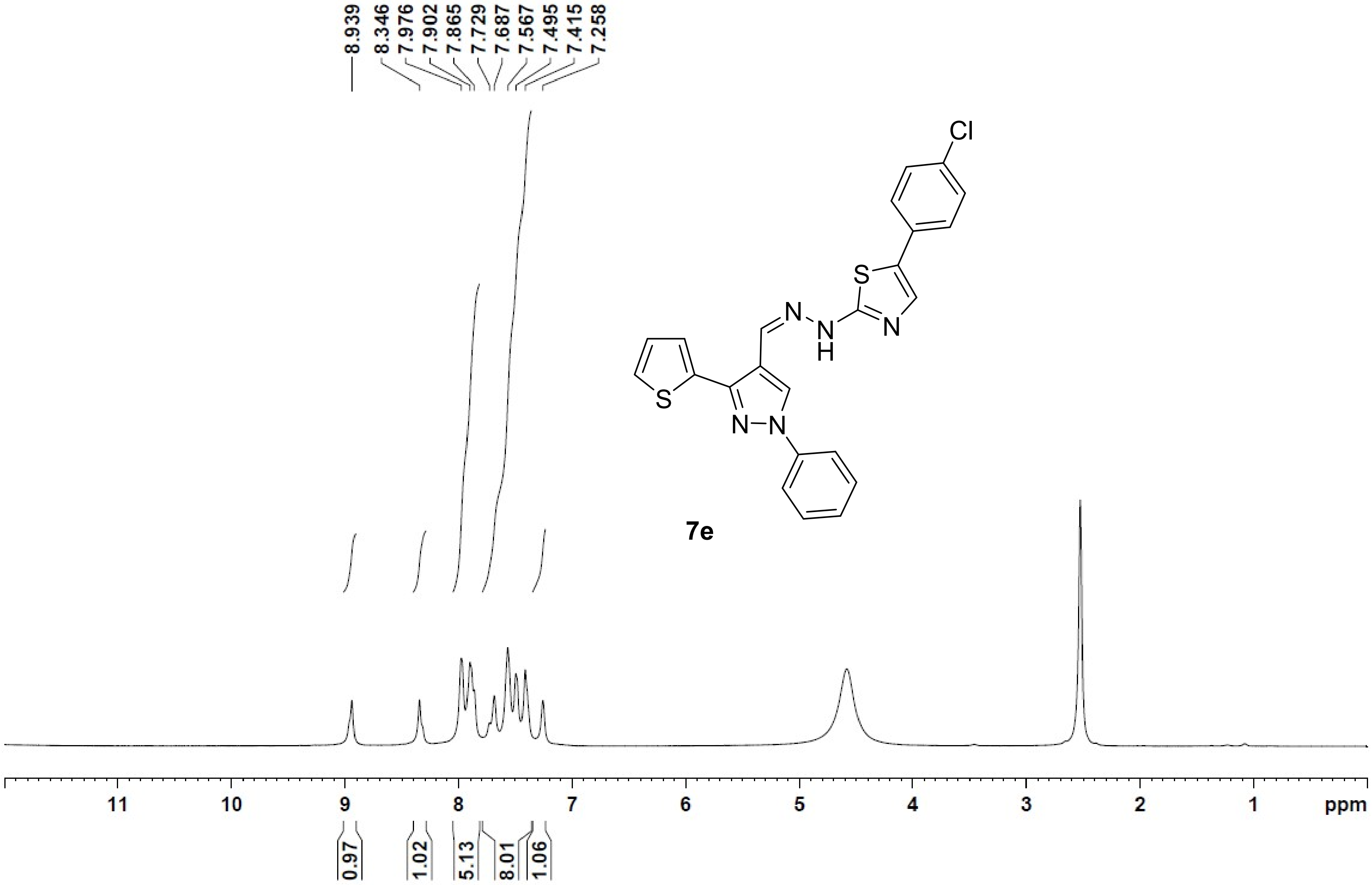
8.939
8.346
7.976
7.902
7.865
7.729
7.687
7.567
7.495
7.415
7.258
Cl
S
N
N
H
N
S
N
N
7e
11
10
9
8
7
6
5
4
3
2
1
ppm
0.97
1.02
5.13
8.01
1.06

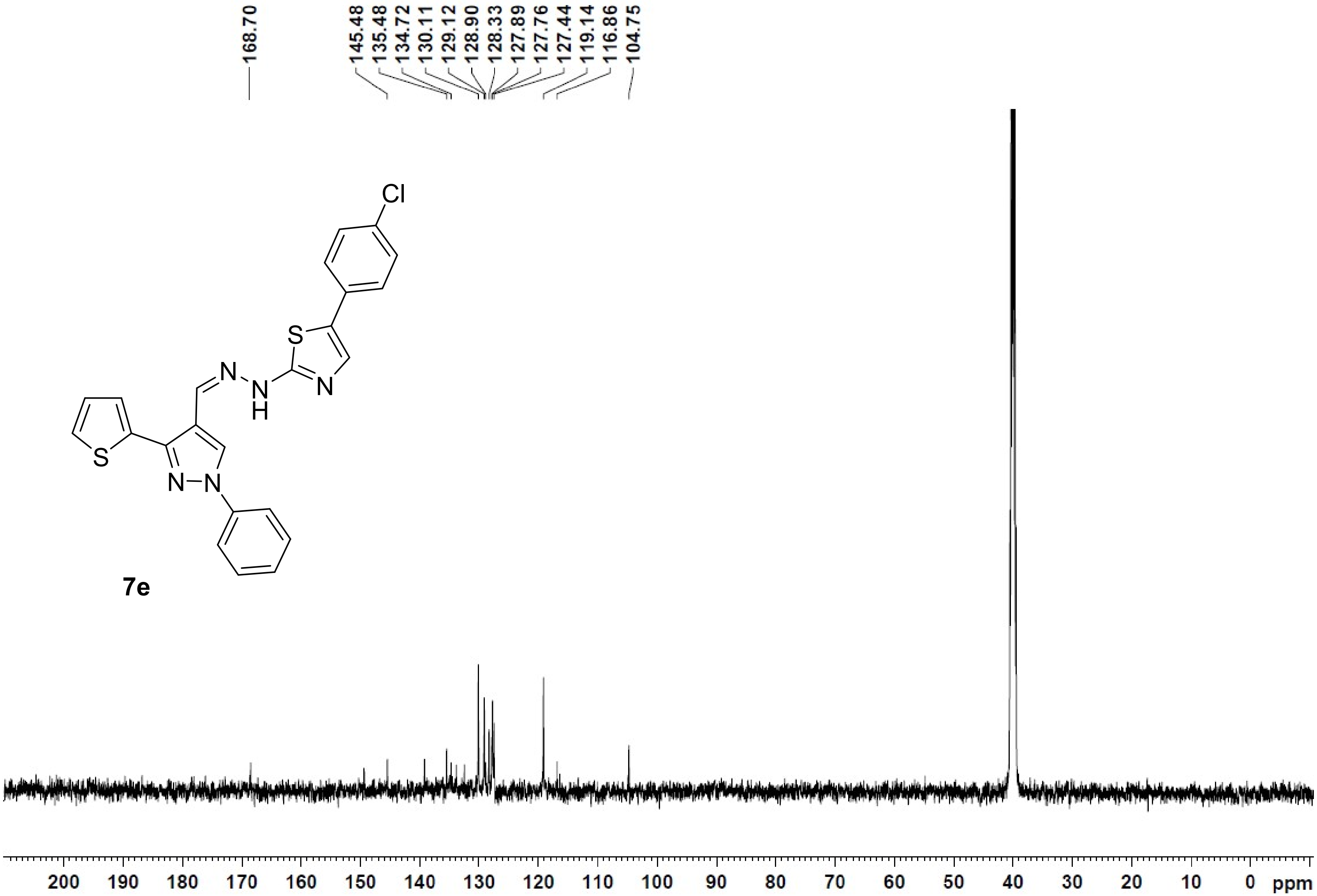
168.70
145.48
135.48
134.72
130.11
129.12
128.90
128.33
127.89
127.76
127.44
119.14
116.86
104.75
Cl
S
N
N
H
S
N
N
N
7e
200 190 180 170 160 150 140 130 120 110 100 90 80 70 60 50 40 30 20 10 0 ppm

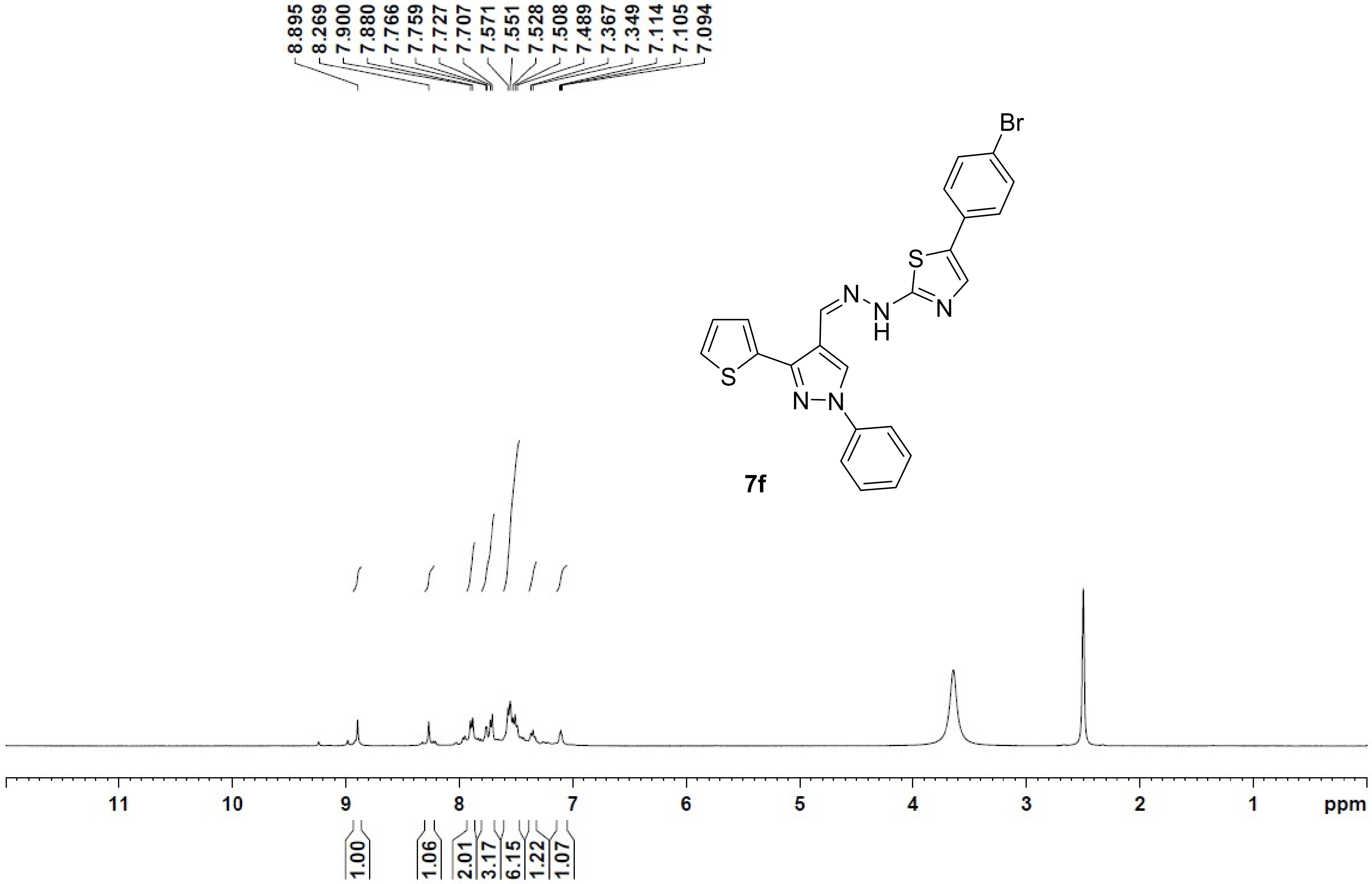
8.895
8.269
7.900
7.880
7.766
7.759
7.727
7.707
7.571
7.551
7.528
7.508
7.489
7.367
7.349
7.114
7.105
7.094
Br
S
N
N
N
H
S
N
N
7f
11
10
9
8
7
6
5
4
3
2
1
ppm
1.00
1.06
2.01
3.17
6.15
1.22
1.07

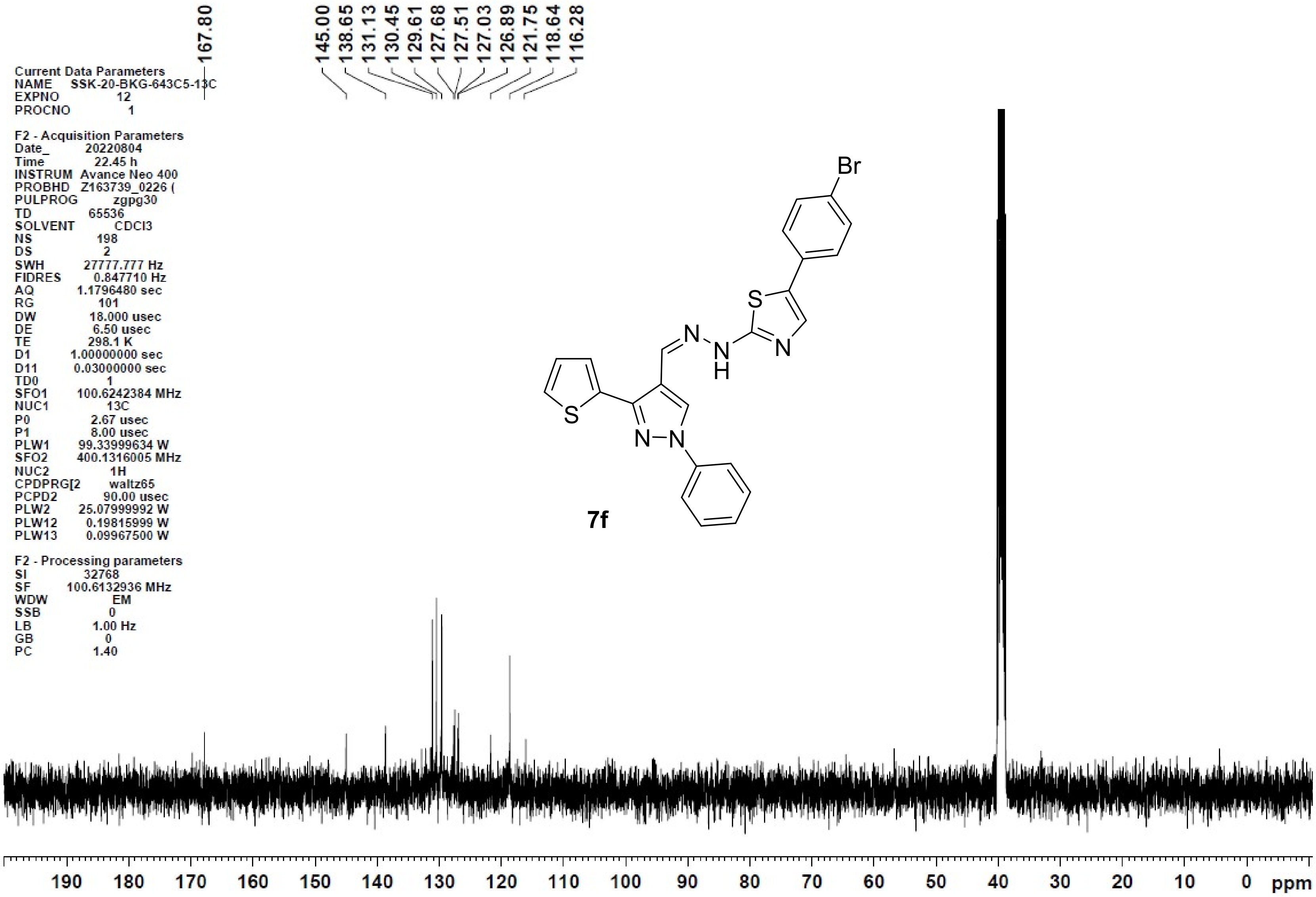
167.80
145.00
138.65
131.13
130.45
129.61
127.68
127.51
127.03
126.89
121.75
118.64
116.28
Current Data Parameters
NAME SSK-20-BKG-643C5-13C
EXPNO 12
PROCNO 1
F2 - Acquisition Parameters
Date_ 20220804
Time 22.45 h
INSTRUM Avance Neo 400
PROBHD Z163739_0226 (
PULPROG zgpg30
TD 65536
SOLVENT CDCl3
NS 198
DS 2
SWH 27777.777 Hz
FIDRES 0.847710 Hz
AQ 1.1796480 sec
RG 101
DW 18.000 usec
DE 6.50 usec
TE 298.1 K
D1 1.00000000 sec
D11 0.03000000 sec
TD0 1
SFO1 100.6242384 MHz
NUC1 13C
P0 2.67 usec
P1 8.00 usec
PLW1 99.33999634 W
SFO2 400.1316005 MHz
NUC2 1H
CPDPRG[2 waltz65
PCPD2 90.00 usec
PLW2 25.07999992 W
PLW12 0.19815999 W
PLW13 0.09967500 W
F2 - Processing parameters
SI 32768
SF 100.6132936 MHz
WDW EM
SSB 0
LB 1.00 Hz
GB 0
PC 1.40
Br
S
N
N
N
H
S
N
N
7f
190 180 170 160 150 140 130 120 110 100 90 80 70 60 50 40 30 20 10 0 ppm

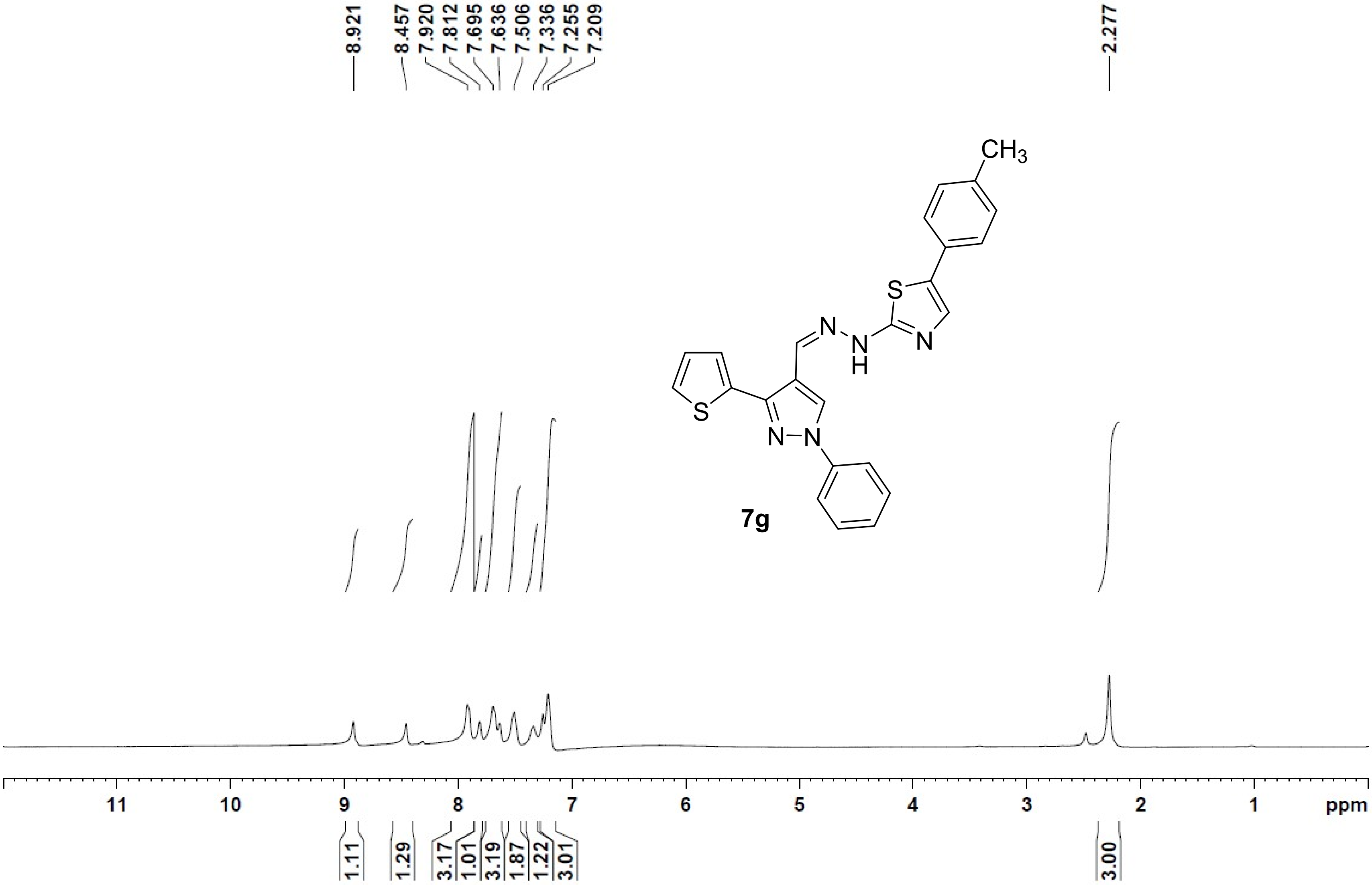
8.921
8.457
7.920
7.812
7.695
7.636
7.506
7.336
7.255
7.209
2.277
CH3
S
N
N
N
H
S
N
N
7g
11
10
9
8
7
6
5
4
3
2
1
ppm
1.11
1.29
3.17
1.01
3.19
1.87
1.22
3.01
3.00

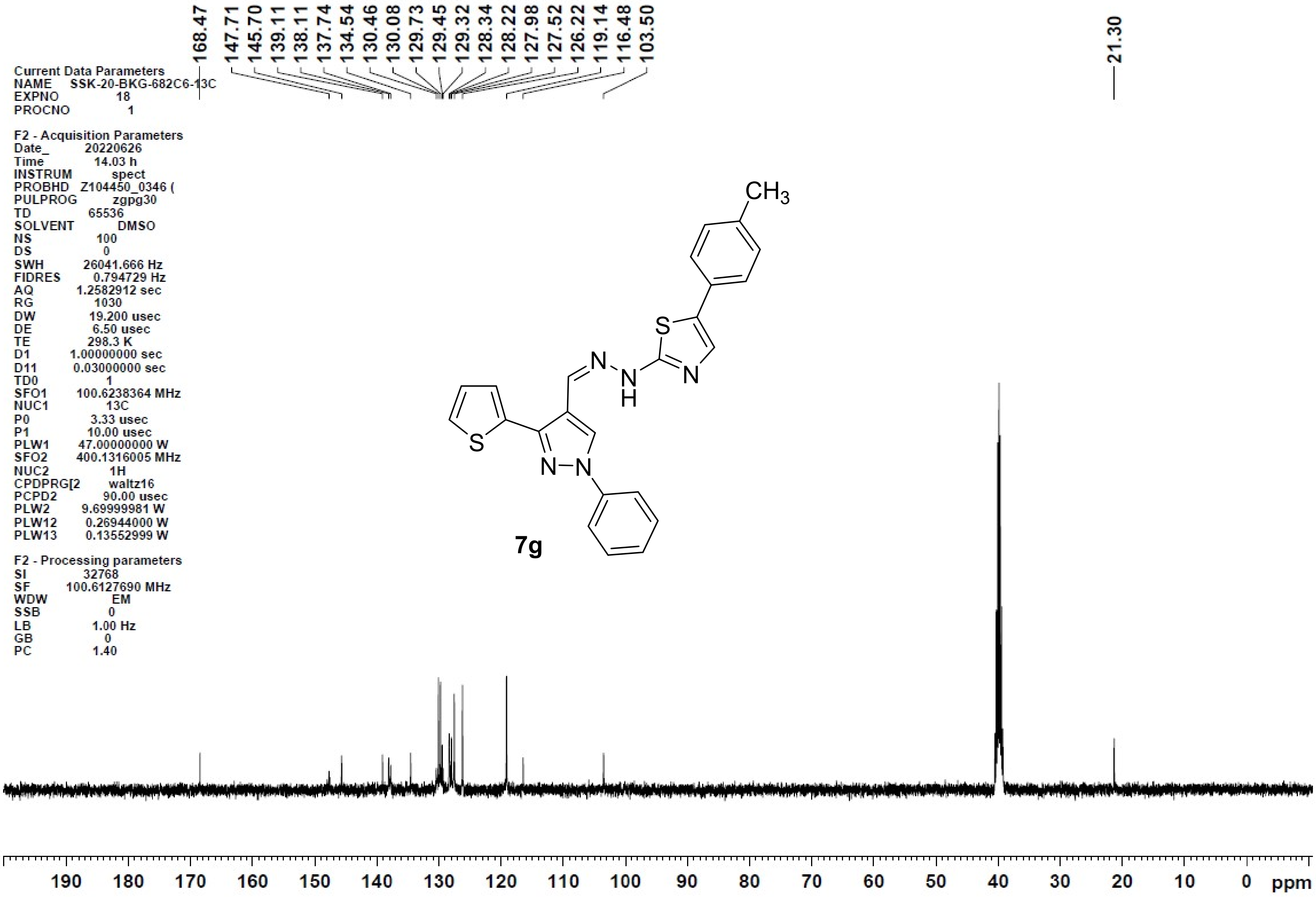
168.47
147.71
145.70
139.11
138.11
137.74
134.54
130.46
130.08
129.73
129.45
129.32
128.34
128.22
127.98
127.52
126.22
119.14
116.48
103.50
21.30
Current Data Parameters
NAME SSK-20-BKG-682C6-13C
EXPNO 18
PROCNO 1
F2 - Acquisition Parameters
Date_ 20220626
Time 14.03 h
INSTRUM spect
PROBHD Z104450_0346 (
PULPROG zgpg30
TD 65536
SOLVENT DMSO
NS 100
DS 0
SWH 26041.666 Hz
FIDRES 0.794729 Hz
AQ 1.2582912 sec
RG 1030
DW 19.200 usec
DE 6.50 usec
TE 298.3 K
D1 1.00000000 sec
D11 0.03000000 sec
TD0 1
SFO1 100.6238364 MHz
NUC1 13C
P0 3.33 usec
P1 10.00 usec
PLW1 47.00000000 W
SFO2 400.1316005 MHz
NUC2 1H
CPDPRG[2 waltz16
PCPD2 90.00 usec
PLW2 9.69999981 W
PLW12 0.26944000 W
PLW13 0.13552999 W
F2 - Processing parameters
SI 32768
SF 100.6127690 MHz
WDW EM
SSB 0
LB 1.00 Hz
GB 0
PC 1.40
CH3
S
N
N
H
N
S
N
N
7g
190 180 170 160 150 140 130 120 110 100 90 80 70 60 50 40 30 20 10 0 ppm

5. References.

[1] G. G. Mandawad, R. D. Kamble, S. V. Hese, R. A. More, R. N. Gacche, K. M. Kodam and B. S. Dawane, An efficient synthesis of isoxazoline libraries of thiophene analogs and its antimycobacterial investigation, *Med. Chem. Res.*, 2014, **23**, 4455. DOI: 10.1007/s00044-014-1016-y.

[2] a) S. S. Mahurkar, S. S. Makone and R. A. More, An efficient and recyclable catalyst for synthesis of 1,3-diphenyl-3-(phenyl thio)propan-1-one derivatives and their antibacterial evaluation, *Chem. Biol. Interact.*, 2019, **9**, 277. b) R. More, G. Sanap, A. Siddiqui, S. Dhutekar, S. Patil, V. Diwan, M. Lokare, A. Ingle and P. Maske, Antibacterial, antioxidant, phytochemicals evaluation of five Indian medicinal plants, *World J. Pharm. Res.*, 2018, **7**, 892.

[3] R. A. More, G. B. Sanap, M. A. Karale, Y. P. Sarnikar and R. N. Gacche, Antioxidant and cytotoxicity profile of the selected alcoholic beverages widely consumed in the Maharashtra State of India, *Indian J. Public Health Res. Dev.*, 2020, **11**, 607.

[4] M. J. Frisch, G. W. Trucks, H. B. Schlegel, G. E. Scuseria, M. A. Robb, J. R. Cheeseman, G. Scalmani, V. Barone, B. Mennucci, G. A. Petersson, H. Nakatsuji, M. Caricato, X. Li, H. P. Hratchian, A. F. Izmaylov, J. Bloino, G. Zheng, J. L. Sonnenberg, M. Hada, M. Ehara, K. Toyota, R. Fukuda, J. Hasegawa, M. Ishida, T. Nakajima, Y. Honda, O. Kitao, H. Nakai, T. Vreven, J. A. Montgomery, Jr., J. E. Peralta, F. Ogliaro, M. Bearpark, J. J. Heyd, E. Brothers, K. N. Kudin, V. N. Staroverov, T. Keith, R. Kobayashi, J. Normand, K. Raghavachari, A. Rendell, J. C. Burant, S. S. Iyengar, J. Tomasi, M. Cossi, N. Rega, J. M. Millam, M. Klene, J. E. Knox, J. B. Cross, V. Bakken, C. Adamo, J. Jaramillo, R. Gomperts, R. E. Stratmann, O. Yazyev, A. J. Austin, R. Cammi, C. Pomelli, J. W. Ochterski, R. L. Martin, K. Morokuma, V. G. Zakrzewski, G. A. Voth, P. Salvador, J. J. Dannenberg, S. Dapprich, A. D. Daniels, O. Farkas, J. B. Foresman, J. V. Ortiz, J. Cioslowski, and D. J. Fox, Gaussian, Inc., Wallingford CT, 2013.

[5] a) A. D. Becke, *J. Chem. Phys.* 1997, **107**, 8554; b) Q. Wu, W. Yang, *J. Chem. Phys.* 2002, **116**, 515; c) S. Grimme, *J. Comput. Chem.* 2006, **27**, 1787; d) J. Da Chai, M. Head-Gordon, *Phys. Chem. Chem. Phys.* 2008, **10**, 6615.

[6] a) A. Schäfer, H. Horn, R. Ahlrichs, *J. Chem. Phys.* 1992, **97**, 2571; b) A. Schäfer, C. Huber, R. Ahlrichs, *J. Chem. Phys.* 1994, **100**, 5829; c) F. Weigend, R. Ahlrichs, *Phys. Chem. Chem. Phys.* 2005, **7**, 3297–3305; d) F. Weigend, *Phys. Chem. Chem. Phys.* 2006, **8**, 1057.

[7] a) L. K. Zaitri, S. M. Mekelleche. DFT and TD-DFT study on quadratic NLO response and optoelectronic activity in novel Y-shaped imidazole-based push-pull chromophores. *J. Mol. Model.* 2021, **27**, 136. DOI: 10.1007/s00894-021-04764-7; b) Becke, A. D. Density-functional thermochemistry. III. The role of exact exchange. *J. Chem. Phy.*, 1993, **98**, 5648. DOI: 10.1063/1.464913; c) Lee, C., Yang, W., & Parr, R. G. Development of the Colle-Salvetti correlation-energy formula into a functional of the electron density. *Physical Review B*, 1988, **37**, 785. DOI: 10.1103/PhysRevB.37.785

[8] A. D. Boese, N. C. Handy, *J. Chem. Phys.* 2002, **116**.

[9] D. Lim and N. C. J. Strynadka, Structural basis for the β lactam resistance of PBP2a from methicillin-resistant Staphylococcus aureus. *Nat. Struct. Mol. Biol.*, 2002, **9**, 870–876, DOI: 10.1038/nsb858.

[10] H. Kishida, S. Unzai, D. I. Roper, A. Lloyd, S. Y. Park and J. R. H. Tame, Crystal Structure of Penicillin Binding Protein 4 (dacB) from Escherichia coli, both in the Native Form and Covalently Linked to Various Antibiotics. *Biochemistry*, 2006, **45**, 783–792, DOI: 10.1021/bi051533t.

[11] E. Sauvage, C. Duez, R. Herman, F. Kerff, S. Petrella, J. W. Anderson, S. A. Adediran, R. F. Pratt, J. Frère and P. Charlier, Crystal structure of the Bacillus subtilis penicillin-binding protein 4a, and its complex with a peptidoglycan mimetic peptide. *J. Mol. Biol.*, 2007, **371**, 528–539, DOI: 10.1016/j.jmb.2007.05.071.

[12] T. Y. Hargrove, L. Friggeri, Z. Wawrzak, A. Qi, W. J. Hoekstra, R. J. Schotzinger, J. D. York, F. P. Guengerich and G. I. Lepesheva, Structural analyses of Candida albicans sterol 14α-demethylase complexed with azole drugs address the molecular basis of azole-mediated inhibition of fungal sterol biosynthesis. *J. Biol. Chem.*, 2017, **292**, 6728–6743, DOI: 10.1074/jbc.M117.778308.

[13] M. V. Keniya, M. Sabherwal, R. K. Wilson, M. A. Woods, A. A. Sagatova, J. D. A. Tyndall and B. C. Monk, Crystal Structures of Full-Length Lanosterol 14α-Demethylases of Prominent Fungal Pathogens Candida albicans and Candida glabrata Provide Tools for Antifungal Discovery. *Antimicrob. Agents Chemother.*, 2018, **62**, e01134-18, DOI: 10.1128/AAC.01134-18.
